# Rubber-like elasticity in laser-driven free surface flow of a Newtonian fluid

**DOI:** 10.1073/pnas.2301956120

**Published:** 2023-06-26

**Authors:** Meghanad Kayanattil, Zhipeng Huang, Djordje Gitaric, Sascha W. Epp

**Affiliations:** ^a^Max Planck Institute for the Structure and Dynamics of Matter, 22761 Hamburg, Germany

**Keywords:** elasticity, Newtonian fluid, laser ablation, bubble, spallation

## Abstract

The energy needed to deform an elastic solid may be recovered, while in Newtonian fluids, like water and glycerol, deformation energy dissipates on timescales of the intermolecular relaxation time $\tau_{M}$ . For times considerably longer than $\tau_{M}$ the existence of shear elasticity requires long-range correlations, which challenge our understanding of the liquid state. We investigated laser-driven free surface bubbles in liquid glycerol by analyzing their expansion and bursting dynamics, in which we found a flow-dominating, rubber-like elasticity unrelated to surface tension forces. In extension to findings of a measurable liquid elasticity at even very low deformation frequencies [L. Noirez, P. Baroni, *J. Mol. Struct.* **972**, 16–21 (2010), A. Zaccone, K. Trachenko, *Proc. Natl. Acad. Sci. U.S.A.* **117**, 19653–19655 (2020)], that is difficult to access under increased strain, we find a robust, strain rate driven elasticity. The recovery of deformation energy allows the bursting bubble to reach Taylor–Culick velocities 20-fold higher than expected. The elasticity is persistent for microseconds, hence four orders of magnitude longer than $\tau_{M}$ . The dynamic shows that this persistence cannot originate from the far tail of a distribution of relaxation times around $\tau_{M}$ but must appear by frustrating the short molecular dissipation. The longer time should be interpreted as a relaxation of collective modes of metastable groups of molecules. With strain rates of 10^6^ s^−1^, we observe a metastable glycerol shell exhibiting a rubber-like solid behavior with similar elasticity values and characteristic tolerance toward large strains, although the molecular interaction is fundamentally different.

## Significance

Upon deformation, Newtonian fluids are expected to exhibit viscous behavior, and only when deformed on very short timescales, below the molecular diffusion time of a single molecule, is a solid-like elastic response expected. We have revealed a strong, rubber-like elasticity in the Newtonian fluid glycerol by analyzing the dynamics of a laser-driven free surface bubble. Not only do we find an elasticity persistent for four orders of magnitude longer than the diffusion time but also observe tolerance to large deformations only found in rubber-like materials. Our observations are independent of surface tension and require the existence of a transient state with solid-like long-range correlations different from the bulk state. This invites us to revisit our understanding of the liquid state.

A shear strain γ in an elastic (1) substance provokes a related stress Σ=Gγ , with elastic shear constant G and an associated force trying to restore the particle positions prior to the straining. A Newtonian fluid (2), on the contrary, reacts with a viscous flow where the strain rate $\dot{\gamma }$ , not the strain, is in the simplest case proportional to the stress $\Sigma =\eta \dot{\gamma }$ with shear viscosity η (3). The viscous matter has hence no “memory” of the shape prior to straining. Contrary to common perception and ordinary practical experience, that liquids are defined by the absence of shear elasticity in contrast to solids, all liquids offer shear elasticity and therefore show non-Newtonian behavior if strained with sufficiently high frequency or on timescales shorter than the relevant mean intermolecular relaxation time $\tau_{M}$ . For small molecular mass liquids, like water and glycerol above their melting points, $\tau_{M}$ is well below the nanosecond (4–9). While at times $t<\tau_{M}$ , Newtonian fluids may show amorphous solid-like elastic behavior, for longer times we experience them liquid-like viscous (2). For $t\gg \tau_{M}$ the existence of shear elasticity requires long range correlations and challenges our understanding of the liquid state (10–13). Nonzero shear elasticity in fluids has been identified by mostly periodic approaches either under infinitesimal strains at high frequencies of GHz down to about 0.1 MHz (4, 5, 8, 14–18) or, remarkably, at very low frequencies with minuscule contributions (19–21).

Here we present the discovery of a rubber-like elastic and large strain response in the Newtonian fluid glycerol, where shear elasticity is not only a measurable contribution but instead dominates the dynamics of the laser-driven free surface bubble for at least several microseconds and four orders of magnitude longer than $\tau_{M}$ . Many bubble phenomena (22) are related to surface tension as the dominating force in static as well as dynamic situations. The elasticity dominating our flow dynamic is not rooted in surface tension invoked forces.

## Results

**Fig. 1.**
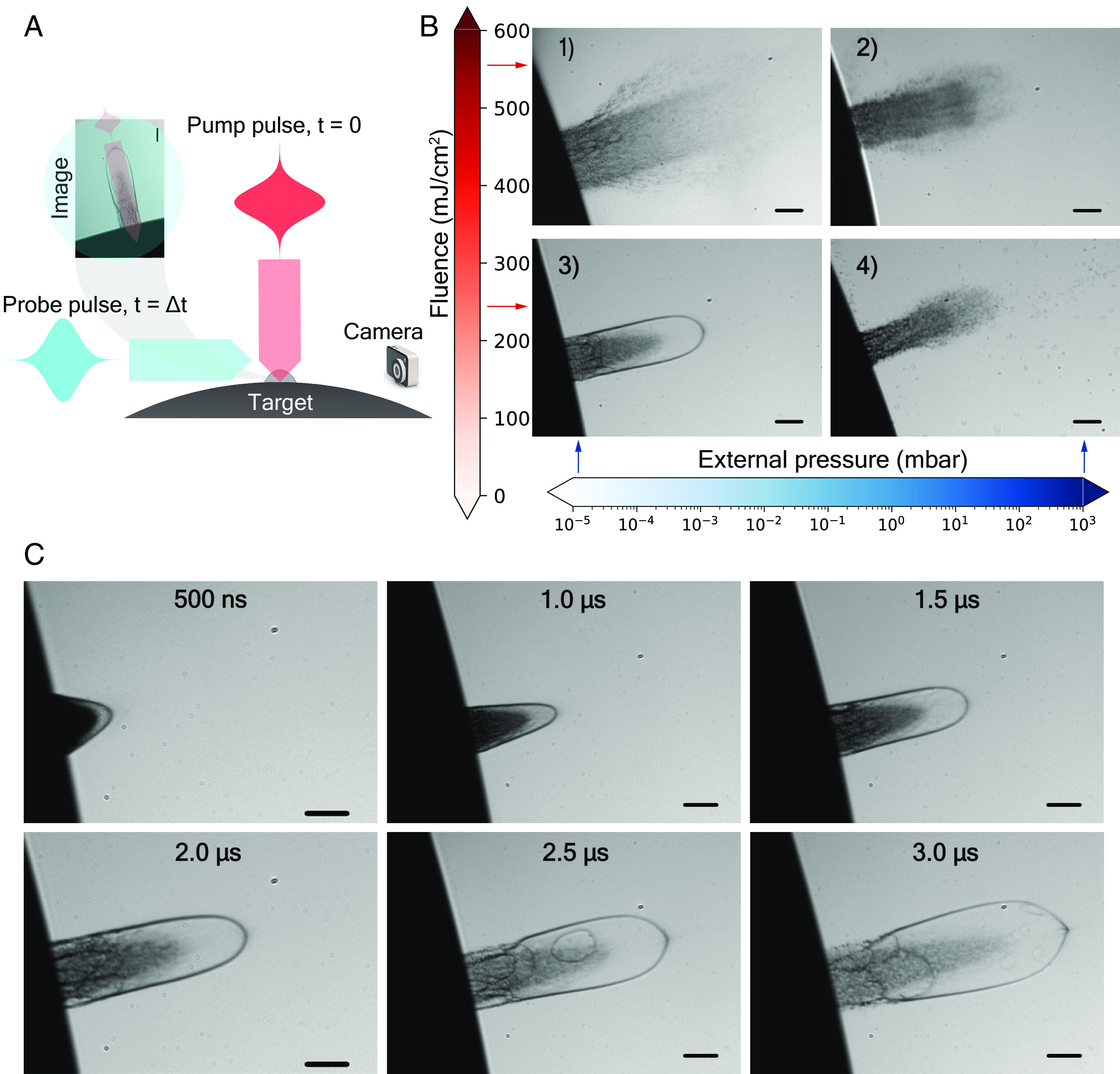
Laser ablation of glycerol in a vacuum. (*A*) Schematic of the ablation and imaging process. The ablation laser (pump) pulse (red) travels top to down, and the probing imaging pulse (blue) travels left to right and records a snapshot of the plume on a CCD camera. (*B*) Ablation plumes at selected fluence (570 ± 34 mJ/cm^2^ and 240 ± 11 mJ/cm^2^) and pressure conditions (1,000 mbar and 10^−5^ mbar) as indicated. The formation of a bubble is very distinct from other conditions. All images are taken at a probe delay of Δt  = 2 μs. The black scale bar corresponds to a length of 100 µm. (*C*) Set of snapshots of different ablation events at different pump delays Δt between 500 ns and 3 µs, as indicated. Each image results from a different pump (ablation laser) and probe (nanosecond discharge lamp) event, not a temporal sequence of the same plume with a single ablation event. Multiple images are taken for any delay value on a statistical basis. The black scale bar corresponds to a length of 100 µm.

Our experimental setup is described in *Materials and Methods* and briefly consists of a vacuum chamber allowing for experiments at different pressure conditions. A subnanosecond pulse-length laser with a central wavelength of 3 μm is coupled into the chamber and focused onto a liquid sample. From a direction perpendicular to both, the surface normal of the liquid as well as the direction of the laser, we capture snapshots by time-resolved brightfield imaging of the dynamic response of the laser-driven liquid at times relative to the arrival of the pulses at the surface (Fig. 1*A*). The experimental parameters we alternate for the data presented here are the fluence of the laser and the pressure conditions of the experiment. Broadly, it is either atmospheric or vacuum conditions. The most relevant results are gained for glycerol under vacuum conditions. It is, however, worthwhile to contrast the dynamics of glycerol and water at atmospheric conditions to appreciate the differences.

For glycerol (Fig. 1*B* panel 2; *SI Appendix*, Fig. S1*B*) and water (*SI Appendix*, Fig. S1*A*), at atmospheric pressure conditions and laser fluences of 550 ± 33 mJ/cm^2^ a well-known plume dynamic unfolds (23, 24) as a consequence of a stress confined absorption (25). Visible is a leading shock front (*SI Appendix*, Fig. S1*A*) trailed by matter in a thermodynamically supercritical mixture of gas and liquid droplets after parts of the heated liquid went through a phase explosion. At present conditions, the absorption length of water is about 1.2 µm (26), and the deposition of nearly the entire pulse energy unfolds along a few micrometers thick layer. Although the absorption length is slightly longer in glycerol (*SI Appendix*, section A), a comparable dynamic evolves based on a similar absorption spectrum caused by similar bonding.

At the heart of our study are the dynamics of liquid glycerol under vacuum conditions (Fig. 1 *B* and *C*), where glycerol at room temperature of 20 °C is, unlike water, sufficiently stable against vaporization to conduct the experiment. For peak fluences of 570 ± 34 mJ/cm^2^ and pressure below 10^−5^ mbar, we find a plume with slowed down dynamic and without the leading air shock front due to vacuum. A remarkable dynamic evolves (Fig. 1*B* panel 3; Fig. 1*C* and Movie S1) when we tune the laser fluence at the half value to $F_{p}=240\;\pm \;11\;\mathrm{mJ}\;/\;{\mathrm{cm}}^{2}$ within a narrow range of 170 to 240 mJ/cm^2^. Now, a bubble with dimensions of hundreds of micrometers is formed with a liquid shell of varying wall thickness on the order of a few micrometers (*Materials and Methods*). For fluences lower than $F_{p}$ , we find plumes with highly defective shells from the onset, and at higher fluences, we see a nebulized remnant of the shell in accordance with Leisner et al. (27) (*SI Appendix*, Fig. S1*C*).

The formation of bubbles or micro bumps is well known and has been studied in the context of laser-induced forward transfer (LIFT) (28) and in laser ablation of metals (29). In the case of metal ablation, depending on the fluence, the process is broadly divided into three regimes; melting, spallation, and phase explosion, where a similar type of bubble formation has been observed in the spallation regime. Bubble formation during LIFT with aqueous glycerol and surfactant mixtures of it has been reported (30, 31), but at smaller sizes and significantly different conditions and driving mechanisms (*SI Appendix*, section D).

Our bubble shell under vacuum conditions is predominantly formed by photomechanical ablation (spallation) (25), which is based on mechanical stress leading to fracture and ejection, ideally in the absence of the phase change observed for other experimental parameters as described above. For spallation, the free surface of the glycerol effectively reflects the laser-generated compression wave. This process adds tensile components to the original compressional components. Once the local tensile strength of the liquid is reached, spallation can develop, separating regions of the liquid (*SI Appendix*, section B and C). This process differs significantly from the LIFT process (*SI Appendix*, section D), which is driven by a pressurized volume of vaporized liquid that is not prominent in our shell dynamics.

### Tip Motion.

**Fig. 2.**
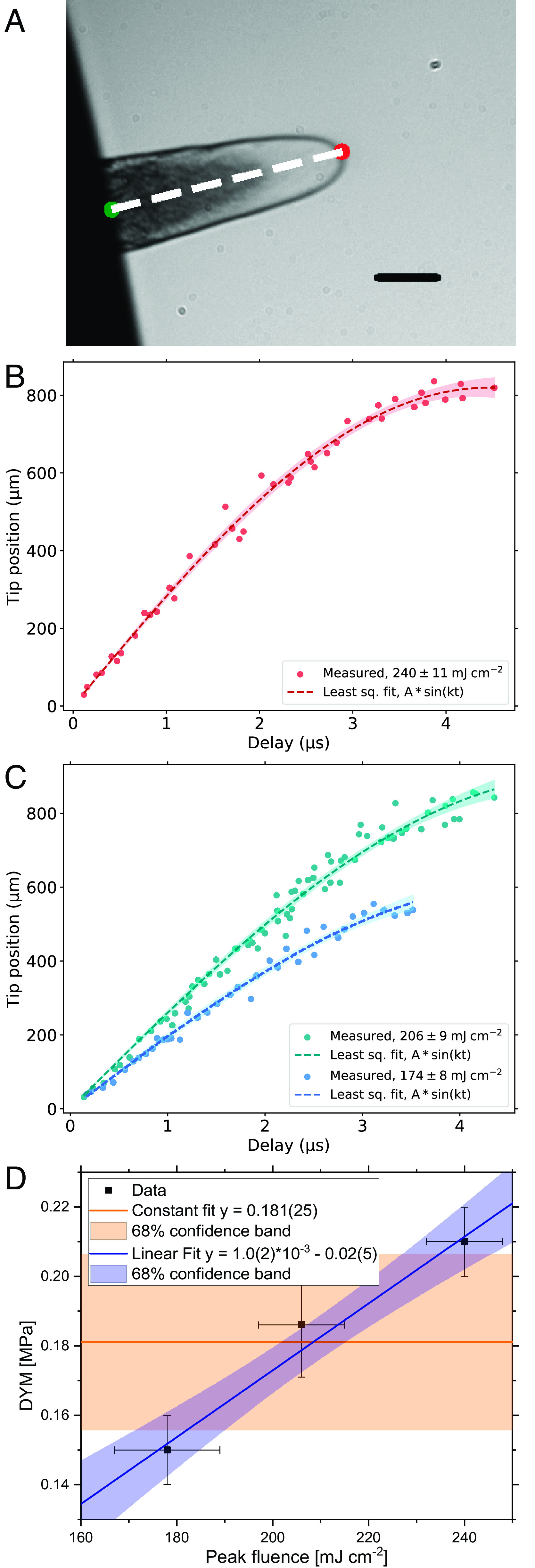
Bubble tip position vs. time. (*A*) For different probe delays Δt=t (here 1.5 µs), the bubble tip position $h_{s}\left(t\right)$ is determined as the respective distance of the tip (red) relative to the bulk (green). The black scale bar corresponds to a length of 100 µm. (*B*) Evaluation of tip positions at different probe delays Δt for high ablation fluence (240 ± 11 mJ/cm^2^). The dashed line is obtained by fitting the measured data points to a sine function hst=hmax∗sin2πT∗t . The shaded area represents the 95% confidence band of the fit. (*C*) Same as *B*, but for the low and medium fluences of 174 ± 8 mJ/cm^2^ and 206 ± 9 mJ/cm^2^, respectively. (*D*) Dynamic Young’s modulus vs. ablation fluence, the data is consistent with Young’s modulus monotonically increasing with increasing fluence. A linear, as well as constant fit is applied to the data.

We will utilize the motion of the tip of the bubble to infer the elastic parameters of the expanding shell. This motion is one-dimensional and reduces the complexity of the dynamic. Bubble growth is initially well described by a biaxial elongation (32), similar to the inflation of a rubber air balloon (33, 34). The velocity of the tip (Fig. 2*A*), which is a mass point δm that can be traced unequivocally, follows for all investigated laser intensities with bubble formation a surprisingly simple functional behavior (Fig. 2 *B* and *C*). Insight can be gained by identifying the functional behavior of h(t), the distance traveled by δm, with the first quarter period of a sinusoidal motion $h_{s}\left(t\right)=h_{max}*\sin\left(\frac{2\pi }{T}*t\right)$ with T being the period length and $h_{\max}$ the amplitude.

A sinusoidal behavior can be motivated by the eigenfunctions of motion for a thin membrane from a simple elastic Hookean material (22). The assumption of an elastic membrane is, of course, in stark contrast to the viscous behavior of a Newtonian fluid that is usually observed. However, Fig. 2 *B* and *C* show fits to the $h_{s}\left(t\right)$ data, with $h_{\max}$ and T as parameters, for three different laser intensities. We find that the sinusoidal description matches the experimental data very well. Within this one-dimensional reduction of the shell motion, we have also fitted the solution of the damped harmonic oscillator with velocity-depended viscous damping analogous to a Kevin–Voigt (1) rheological model (*SI Appendix*, section E). We expect that the normally dissipative viscous flow of liquid glycerol will dampen the motion. However, we find a reproduction of the pure sine fit and rejection of such a damping model by obtaining tiny damping constants.

Using a procedure described in *Materials and Methods*, we inferred an elastic modulus, here Young’s modulus, from the fit parameters (*SI Appendix*, Table S1). This dynamic Young’s modulus (DYM) is on the order of 0.2 MPa, a remarkably high, rubber-like value. Due to the one-dimensional description, this modulus resembles a spatial average over the bubble. Fig. 2*D* shows the DYM vs. the peak fluence. The behavior of the DYM of the laser-driven liquid shell is consistent with a linear dependence on the laser fluence and hence straining rate $\dot{\gamma }$ , which is up to 3 * 10^6^ s^−1^. A higher straining rate leads to a larger value for the DYM. In experiments of shocked free liquid surfaces, it was found (35) that the tensile strength, the dynamic viscosity, and therefore the relaxation times increase with the strain rate. Eventually, the values plateau at solid-like values, particularly for the dynamic viscosity. A clear understanding is still lacking, but as a side note, it seems reasonable to suspect similar atomistic origins for these and our observations with relevant similarities.

We demonstrated that the motion of the mass point at the tip of the highly strained shell is reproducible by the motion of a linear elastic membrane based on an averaged DYM. While solid elasticity is expressed typically for infinitesimal strains, this elasticity, on the contrary, happens under conditions of severe strain. To our surprise, the motion of the mass point does not call for a more complex, viscoelastic model (2). For our experimental parameters, we calculate a large capillary number of $C_{\mathrm{PL}}\approx 6,000$ and a moderate Reynolds number $R_{\mathrm{PL}}\approx 18$ (*SI Appendix*, section F), which nominally implies that viscous forces dominate over capillary and inertial forces. However, the typical characteristic of energy dissipation attributed to viscous motion appears to be absent in the results. Instead, we find the elastic manifestation of viscosity as formulated by Maxwell (1) with $\eta =\tau_{M}*G$ , which relates viscosity to the elastic constant and the relaxation timescale $\tau_{M}$ . Our data require a $\tau_{M}$ of several microseconds, hence orders of magnitude longer than structural relaxations. Furthermore, it is in line with a dominating single and long relaxation time $\tau_{M}$ and defies the presence of considerable dissipation via other shorter relaxations (14, 17, 18) as it could be the case for models with a spectrum of structural relaxation time scales as the generalized Maxwell model (*SI Appendix*, section E) or others (1–4, 32). The presence of shorter relaxation times might still produce a motion of δm appearing sine-like (*SI Appendix*, section G) on quarter-period observation. However, the dissipative nature would drain the shell’s deformation energy, which contradicts our observation.

A liquid mass transport toward the tip is identifiable in the shell at time points after the shell rupture close to the bulk (lift-off) by a darkening of the shell toward the tip region (*SI Appendix*, Fig. S2). Such transport of liquid is also described for the LIFT process (28) based on a pressure gradient. In contrast, we believe this mass transport also corroborates the diminished role of viscous energy dissipation since it is here a consequence of elastic energy stored in the shell, similar to an inflated rubber balloon (33), which is popped at one pole and the shell is drawn to the opposite pole of the sphere. While all lateral momentum components that are in the plane of the bulk surface cancel for the rotationally symmetric influx to the tip, the unbalanced z components culminate in a narrow jetting in the z-direction (*SI Appendix*, Fig. S2 *A* and *B*).

The onset of lift-off (*SI Appendix*, section H) is a frequently occurring defect to the integrity of the shell (*Materials and Methods*), even at the earlier stages of the dynamic (Fig. 1*C*). The narrow statistical scattering of the data for the tip position, as evident from Fig. 2 *B* and *C*, shows that this statistically appearing defect is of little influence to the dynamic of the tip. The initial dynamic of the tip and the loading of the shell with elastic energy is influenced by the inertia of the slower parts of the shell and the velocity differences between different sections. For the short observation window of the entire dynamic, a perfect anchoring of the shell to the bulk, unaffected by defects, is not necessary to find the observed dynamic.

### Analysis of Hole Growth.

**Fig. 3.**
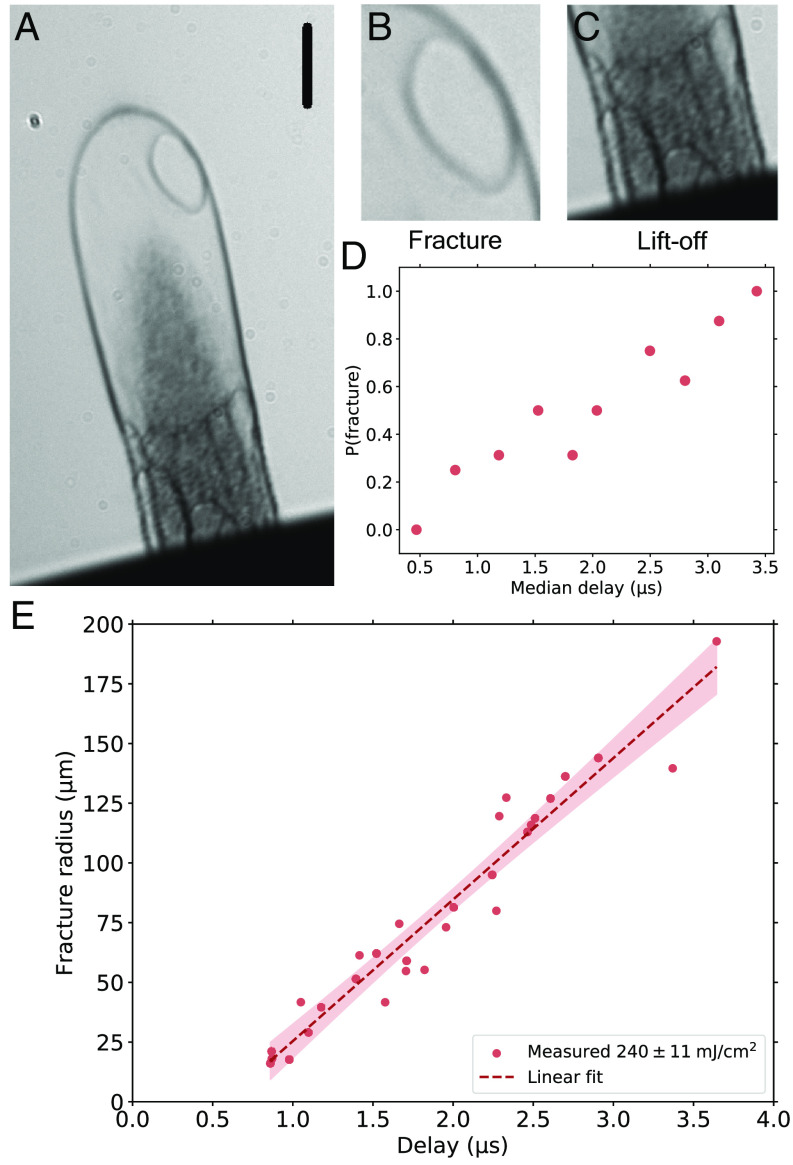
Bubble fracture radius vs. time and fracture probability. (*A*) Snapshot at Δt ≈ 2 μs capturing an ongoing hole expansion, see panel *B*. At the same time, several hole fractures are visible in the shell close to the bulk. The black scale bar corresponds to a length of 100 µm. (*B*) Zoom-in of the hole depicted in panel *A*. The term “fracture” describes the bubble fractures happening at the *Top*. (*C*) Zoom-in to the bulk-shell region as depicted in panel *A*. Lift-off is used to describe the fracture between the thin shell and the bulk liquid. (*D*) Probability of fracture vs. median delay estimated for a set of images. The fracture probability approaches one at a delay of 3.5 µs and longer. This data was recorded for an ablation pulse fluence of 240 ± 11 mJ/cm^2^. (*E*) The radius of the hole fracture for a set of snapshots at different delays. The dashed red line represents the linear least square fit for the scattered values. All measurements are done for an ablation pulse fluence of 240 ± 11 mJ/cm^2^. The shaded area represents the 95% confidence band of the fit.

In a second approach to determine the elastic contributions to the dynamic, especially when the expansion of the shell essentially slowed down, we investigate the hole growth (36, 37) once the shell starts to rupture at time points t > 1 µs (Fig. 3). Culick (38) found that in liquids with low viscosity, the hole expansion during bubble rupture progresses with a constant speed $v_{0}$ , the Taylor-Culick velocity (TCV), as a function of surface tension σ, film thickness δ , and density ρ by $v_{0}=\sqrt{2\sigma /(\delta \rho )}$ (39). The validity of the TCV has been verified by experiments for water and soap films (40). For a thickness range of 5 to 15 µm glycerol film at ambient conditions, the TCV varies between v_0, Gly_ = 4.5 to 2.6 m/s. The viscosity of glycerol does not enter the calculation of the rim velocity, neither theoretically nor experimentally (39). However, for very viscid films, a different behavior was reported (41, 42). Most strikingly, the hole radius develops exponentially with time, and the TCV is now the terminal velocity to be reached for long times. Furthermore, the liquid of the vanished surface does not accumulate as a toroidal structure representing the traveling rim, as found for inviscid films, but gets distributed over the remaining intact surface.

We studied the hole growth in the upper half of the plume (*Materials and Methods*). Due to our stroboscopic recording, we do not have access to the evolution of a particular individual shell, but rather a statistical set of various individual plumes recorded at different time points. Fig. 3*E* depicts the hole radius as a function of the time since the plume was launched. We find a velocity of $v_{0,\;\mathrm{ex}p}=60\pm 3$ m/s for the expansion velocity of the hole radius and from the intercept with the time axis, the statistical earliest rupture birth time at $t_{b}\;=\;0.57\;\pm \;0.11$  μs. The linear fit is consistent with the TCV dependence found in inviscid liquids. In our situation, $v_{0,\;\mathrm{ex}p}$ might potentially underestimate the TCV because the linear fit inherently incorporates the birth of all holes at the time $t_{b}$.

Our TCV ( $v_{0,\;\mathrm{ex}p}$ ) is at least about 20 times larger than the value given by $v_{0,\;\mathrm{Gly}}$ , which is for a viscous Newtonian fluid with the well-known bulk parameters of glycerol (surface tension, σ=63.4  mN/m). This significant deviation either requires the presence of an unaccounted-for force, such as shear elasticity, or the significant underestimation of the actual film thickness together with orders of magnitude deviations from bulk values for one or more parameters like density and surface tension.

Elasticity has been added to Culick’s work (38) in several contexts (43–45), as we detail in *Materials and Methods*. Sabadini et al. (44) recorded rim speeds in bursting soap bubbles containing wormlike micelles (WLM) up to 30 times the TCV but on a dynamics time scale three orders of magnitude longer than ours. They attributed this significant enhancement to the elasticity stored in the WLM during the bubble’s inflation, readily available at rupture to accelerate the process. Recently, Tammaro et al. (45) pointed out the importance of the inflation history, which of course becomes essential when the timescale of the deformation energy relaxation processes is on the order of the observation time and thus affects the TCV.

Again assuming an elastic behavior (*Materials and Methods*), we can equate the elastic constant, here the transient shear modulus G(t) (15), to our hole velocity and the parameters of the experiment:

$$G≅v^{2}*\frac{2\rho }{{\lambda_{1}}^{2}+{\lambda_{2}}^{2}-3}.$$

With strain ratios ${\lambda_{1}}^{2}\approx {\lambda_{2}}^{2}\approx 11$ , derived from simple geometric considerations (*Materials and Methods*), ρ ≈ 1,260 kg/m^3^ and m/s $v=v_{0,\;\mathrm{ex}p}=60$ , we find $G=G_{\exp}=0.48\;\mathrm{MPa}$ , which again reveals a surprisingly high elasticity with an elastic constant comparable to natural rubber’s [cis-1,4-polyisoprene (PI)] infinitesimal strain storage modulus $G^{\prime }\left(\omega \right)$ (up to kHz frequency) (46) (*SI Appendix*, section I). In more relevant large strain experiments with strips of PI elongated to values around $\lambda_{\mathrm{PI}}-1=\varepsilon_{\mathrm{PI}}=2.3$ and the subsequent free movement of the loose end toward the fixed end of the strip, Bogoslovov et al. (47) derived mechanical properties of PI (48) and a large strain shear modulus $G_{>PI}$ , almost four times smaller than our $G_{\exp}$ in laser-driven liquid glycerol.

## Discussion

By analyzing the tip motion and, complementary, the rupture process dynamics, we determined rubber-like elastic constants as measures of elasticity in glycerol. A surprisingly simple elastic behavior on the microsecond time scale for large strains of the order of several hundred percent and strain rates of about 3 * 10^6^ s^−1^ is found in this laser-driven free surface flow. In such a regime, glycerol appears to be the simplest example of a simple viscoelastic fluid (49): An elastic solid modified so that the effect of a strain on the stress is an exponentially decaying function of the time since the strain occurred. Under our conditions, the metastable elastic state lasts at least microseconds and four orders of magnitude longer than the structural relaxation times. Shorter relaxation channels contributing to viscous dissipation appear to be of secondary importance over the bubble's lifetime. The simplest macroscopic model explaining the dynamic is an adjusted Maxwell-like model $\eta_{A}=\tau_{A}*G^{\prime }$ with dynamic viscosity $\eta_{A}$ and a $G^{\prime }\ll G_{\infty }$ with $G_{\infty }=\eta /\tau_{M}$ being the instantaneous shear modulus (15). This again resembles a pure viscous dynamic, where only the elastic manifestation is observable due to the enhanced relaxation time $\tau_{A}$ , which is much longer than the nominal structural relaxation times. We do not have direct access to the value of $\tau_{A}$ , but our observations would suggest the order of 10 µs and above. According to Maxwell’s equation, the validity of which is debated (12), this implies dynamic viscosity values above 5 Pas or at least 3.5 times the static value. On the other hand, this increase in viscosity cannot be too significant (*SI Appendix*, section J) or over 5- to 10-fold since we still observe the hole opening dynamics of the nature of inviscid fluids. First, because the vacated volume of the hole is collected in the torus of the moving rim (50), and second, because the rim moves at a constant speed.

While the thickness of the shell at any given point on the shell is subject to change with time, our analysis (*Materials and Methods* and *SI Appendix*, Figs. S3, S4, and S5) shows the thickness is well above 1 µm, with most likely values more between 3 and 20 µm throughout the dynamics. Furthermore, such film thicknesses are above the range where the physics of nanoconfined liquids (51) with significantly unpredictable consequences on the physical parameters sets in. Nevertheless, the few micrometer shell is well within the boundaries of the theory put forward by Zaccone et al. (13), where for the limit of zero frequency, a scaling of $G^{\prime }\left(\omega \right)$ with $L^{-3}$ is proposed, with L being the typical confinement length. For our thickness dimensions, a shear modulus of about 1 MPa is predicted for a particular liquid polymer. Although this is a different substance, the theory is potentially close in magnitude to our findings in glycerol. This theory finds support in experiments at very low frequencies (20), where a decreasing elastic constant with increasing strain is reported, and the elasticity is generally very fragile concerning strain and strain rate. The elasticity based on confinement potentially offers an explanation for the findings also at our very different high strain rate conditions. The underlying suppression of low-frequency modes responsible for the soft mechanical response due to confinement is of broad scope. There are no indications that the measurements at low frequency and our findings are in contradiction although taken under very different conditions.

In dynamic situations, such as rapid straining, characteristic parameters can have transient values that differ from bulk values. In the case of surface tension (52), a more than 300-fold increase would be required for a transient surface tension to explain our observed TCV. Interestingly, for thin shells, an existing shear elasticity could be experienced as apparent surface tension (32) (*Materials and Methods*), and shear elasticity may be difficult to disentangle from surface tension, although of very different origin. Here, distinguishing surface tension effects from shear elasticity is accomplished by again analyzing the motion of δm. While conversion of the initial pure kinetic energy $E_{v}$ of the shell into elastic energy $E_{G}$ or, in the competing scenario, into surface energy $E_{\sigma }$ is likewise possible, the dynamic must be different for the two possible scenarios, and in comparison, it would hold $E_{G}\left(t\right)\neq E_{\sigma }\left(t\right)$ for most times except t=0 and $t=t^{\prime }$ with $E_{v}\left(t^{\prime }\right)=0$ . Therefore, the scenario with surface energy as the dominant sink of kinetic energy would not reproduce the motion of the tip observed according to Fig. 2 (*Materials and Methods*).

Due to the transient nature of the bubble dynamics on the order of microseconds, the characterization of the dynamic by dimensionless flow numbers (*SI Appendix*, section F) might be of limited insight. An elastocapilary number $E_{c}$ (45) of the form ${{E_{c}=r}_{B}*G}_{\exp}/\sigma \approx 600$ can be introduced reflecting the dominance of elastic forces over capillary forces in the short observation window by relating the bubble size $r_{B}$ , the experimental elastic shear modulus and the surface tension (*SI Appendix*, section F). If we utilize the close entanglement of surface tension effects and shear elastic effects on the dynamic of the shell, we could express the elastic effects, although of very different origin, as an apparent surface tension $\sigma_{\mathrm{ap}}$ about 320 times larger than σ (*SI Appendix*, Eq. **S14**). This leads to an apparent capillary number $Ca_{\mathrm{ap}}$ , which is accordingly 320 times smaller than Ca and reflects the diminished role of viscous forces in the initial dynamics of this bubbles.

Our results provide evidence of a strong, yet unaccounted-for, transient rubber-like elasticity in glycerol. Certainly, glycerol as a relatively small molecule does not possess the entangled molecular chains responsible for the elasticity in rubber (32). Glycerol is a glass former when cooled down to 220 K and possesses a hydrogen bond network relevant for elasticity (53). The physics of the liquid-glass transition (54) (LGT) is studied extensively. Dramatic changes in thermodynamics and dynamics within a narrow temperature range have to be explained, while the changes in structural particle positions are subtle. Toward the LGT one finds increasing relaxation times as well as increasing shear elasticity. This increase of the very observables is in some analogy to the characteristics of our glycerol bubble. Toward the LGT also the viscosity will dramatically increase following the exponential Vogel–Fulcher–Tammann equation (54) for supercooled liquids. The liquid forming our shell is not supercooled since the experiment was performed at room temperature, and additional optical energy absorption further increased the temperature. An adiabatic formation of cold and warm temperature bands within strained glycerol has been reported (55) but is presumably of little relevance to the thermodynamic state at the given energy balance. The striking increase in relaxation time requires structures within the fluid with related and equally large relaxation times. Instead of single molecules subject to fast diffusion, groups of molecules must form that are displaced in a correlated and collective manner (12, 17, 18, 35), thus providing much longer time constants than the single molecule. Understanding these collective excitations in disordered systems is of vital importance. Our experiment invites us to rethink these correlations and the differences between liquids and solids. Unfortunately, our experimental technique does not provide access to the molecular origins or atomistic details of the observed unique dynamics.

Therefore, future experiments will focus on atomic structure investigations of the transient elastic shell and the generality of the effect in various liquids of different intermolecular interactions. The creation of elastic bubbles in water would be of paramount importance. Our study shows that the vacuum condition is a crucial prerequisite for forming such elastic bubbles. Due to the lower vapor pressure, vacuum conditions were so far realizable for glycerol but not for liquid water, which starts boiling below the vapor pressure of 32 mbar.

In addition to their impact on the current understanding of the liquid state and elasticity in fluids, these results could be likewise relevant in a technological or biological background where the timescales for this metastable state are potentially accessible and glycerol is a fundamental component.

## Materials and Methods

### Setup and Ablation Imaging Measurements.

The setup is depicted in *SI Appendix*, Fig. S6, and consists of a vacuum chamber housing the interaction region, where a laser interacts with the sample, held at room temperature, forming a plume. The sample dynamics upon laser interaction are imaged in temporally resolved experiments.

We used water (Milli-Q, Merck, Germany) for atmospheric ablation and glycerol (≥99.5%, Merck (Sigma-Aldrich), Germany) for atmospheric as well as vacuum ablation. The pressure of the experiment between atmospheric and 10^−5^ mbar was controlled by a leak valve and the pumping system (Edwards Vacuum, UK; Pfeiffer Vacuum, Germany). A glycerol droplet lasts several thousands of ablation shots and is stable in a vacuum for hours.

The pulsed ablation laser (Light-Matter Interaction, Canada) emits at a wavelength of $\lambda_{L}=\left(2.92\pm 0.01\right)$ µm and a pulse length of $\tau_{L}=\left(400\pm 50\right)\;\mathrm{ps}$ . After spatial filtering to improve beam quality, the energy per pulse is up to 120 µJ or less by inserting neutral density filters (Thorlabs, USA). The experiments were performed at different energies and pressure levels. Most relevant, in the bubble forming regime, at three different pulse energy levels, high, medium, and low energy around 53, 45, and 38 µJ, respectively. This energy is focused on the interaction point using a focusing lens (f = 300 mm) placed outside the experimental chamber. The FWHM of the beam is about 139 µm with a reasonably Gaussian beam profile recorded with a beam profiler (WinCamD, DataRay Inc.). Laser peak fluences of 240 ± 11, 206 ± 9, and 174 ± 8 mJ/cm^2^ are found for the three pulse energy levels used for bubble formation.

A triggerable discharge arc lamp (Nanolite, Germany) with 12 nanosecond pulse duration was used as the illumination source for imaging the ablation plumes at different time points. A lens (f = 60 mm) collects parts of the light emission, and a field lens (f = 400 mm) focuses the light pulse onto an adjustable iris upstream of the plume. Subsequently, the pulse is condensed by a condenser lens (f = 80 mm). The illumination path was adjusted so that the ablation was in the condenser focus plane to achieve the best contrast. An objective lens with a focal length of 65 mm was used to produce an intermediate image. This image was further projected using a long working distance microscope (Qioptic, UK) with a 200 mm focal length resulting in a final 4× magnification onto a monochrome CCD camera (DMK 23U274, Imaging Source, Germany) with (1,600 * 1,200) pixels.

The ablation laser, flash lamp, and CCD camera were synchronized and provided the temporal information typical to a pump-probe measurement. Using a delay generator (Quantum Composer Inc., USA), the ablation laser triggered the discharge lamp. The CCD camera was also coarsely synchronized with the laser to capture the ablation event within the 0.5 ms acquisition time of the CCD. A shutter synchronized with the ablation laser was used to reduce the repetition rate of the ablation laser from 1 KHz to 1 Hz at the sample position. The timing information is provided solely by the adjusted delay between the ablation laser and the illumination arc lamp. Two photodiodes were used to precisely determine this delay by recording the voltage signal on a digital oscilloscope (Keysight Technologies, USA) on a shot-to-shot basis. Each plume image is stored with a time stamp, the ablation laser pulse energy, and the measured delay value.

The melting point of glycerol is between 291 and 293 K, and it is well known to exist in a supercooled liquid form at much lower temperatures. Non-Newtonian behavior has been reported due to heterogeneity at these supercooled temperatures (56, 57). To eliminate the influence of a supercooled state, we applied a pre-heating pulse from the ablation laser of the same pulse energy one millisecond before the observed ablation at the exact location. In addition, the creation of a fresh sample surface by this pre-pulse prior to each recorded plume improves the stability and quality of the bubble.

### Shell Thickness Estimates.

We find the shell thickness from the optical light absorption behavior of the shell deducible from our recorded brightfield images, which are only two-dimensional intensity representations of a three-dimensional absorption object.

Generally, optical path length in systems with pure absorption can be deduced using the Beer-Lambert law:

[1] $$\log\left(\frac{I}{I_{0}}\right)=A=\varepsilon Zc.$$

With concentration c, optical path length z, absorptivity ε , and the intensities before and after the absorption $I_{0},\;I$ , respectively. Re-writing Eq. **1** gives:

[2] $$I=I_{0}\;\exp\left(-aZ\right).$$

While a is the constant that includes contributions from the concentration and absorption properties of the material, deviations from the linear path length dependence of the exponent can occur when a light source is not monochromatic, the concentrations of the analytes are very high, or when the medium is highly scattering (58, 59). In the case of brightfield imaging, the process involved in the image formation is more complex and includes absorption, scattering, and refraction, and Eq. **2** might be insufficient to apply.

We had success in using a more general form of Eq. **2**, which is, however, entirely phenomenological:

[3] $$I\left(Z_{e}\right)=I_{1}+I_{0}\;\exp\left(-aZ_{e}^{b}\right).$$

With additional parameters $I_{1},\;b$ introducing a nonlinear behavior in the exponent. Once fully defined, Eq. **3** gives the functional relationship between a local intensity in a plume image and the effective path length $Z_{e}$ , which must be interpreted as the path length in liquid glycerol at normal density and temperature. The assumption that the shell is liquid and at normal density rests on the incompressibility of glycerol and is essential for the thickness determination.

The procedure to determine the parameters and finally obtaining a function $I(z_{e})$ for the optical path length through the plume is as follows: First, very early plumes with a delay of less than 200 ns are identified. For these plumes, see *SI Appendix*, Fig. S3, we expect two crucial assumptions to hold. The first is that the structure has rotational symmetry around the z-axis. The second is that the density in these early plumes is constant and equal to (or sufficiently close to) normal bulk density. This is a reasonable assumption for these nascent spalls, which are beginning to detach from the liquid surface. Then for these early plumes, the effective path length for any ray passing through can be found, and to any set of local intensities I of a plume image, a comparable set of effective path length $z_{e}$ is available, retrieved by an iterative process, allowing to fit the parameters of Eq. **3**, see *SI Appendix*, Fig. S3*F*. In practice, we take the local intensity profiles along the white lines as shown in *SI Appendix*, Fig. S3*A*. The z-position of the line is found by examining the intensities along the perpendicular red path parallel to the z-axis and taking the position where the intensity reaches zero or 100 percent absorption. The absorbing object is then equivalent to a solid disc (cylinder of infinitesimal height) of uniform density, see *SI Appendix*, Fig. S3*D*, and we find 100% absorption only along the longest path through the center of the disc.

Secondly, the fully determined Eq. **3** is used for later plumes with a shell structure. Similarly, normalized intensity profiles along the white lines are evaluated, as shown in *SI Appendix,* Fig. S4. The assumed absorption object is now a cylindrical shell according to *SI Appendix*, Fig. S4*D*. We account for our limited optical resolution by convoluting the generated intensity profile with a Gaussian point-spread function of FWHM = 4.7 µm as determined by imaging a calibrated resolution target. The best fit for the bubble shell is determined by iteration over the shell thickness and cylinder diameter parameters. The material ejections visible inside the volume enclosed by the shell are not taken into account, but are considered to be void while creating the geometric path length of the absorption object. The determined shell thickness, subject to an estimated 20% relative error, plotted against the profile distance from the sample surface is shown in *SI Appendix*, Fig. S5. At times around 2 µs, when the shell is well developed and holes with traveling rims begin to form at the top part, we find a thickness distribution as depicted, where the shell at the top has about 50% of the thickness at the bottom, and the thickness increases monotonically from top to bottom. It is unclear whether these thickness differences are a consequence of the shell dynamics or already a consequence of the laser energy distribution, which shows more energy in the center than toward the wings of the beam. This thickness behavior would be consistent with the theoretical solutions we have derived for spall fracture depths that affect shell thickness based on the one-dimensional solution for the thermoelastic wave equation (*SI Appendix*, section C).

### Simulation of the Motion of the Tip Mass Point.

We have simulated the behavior of a linear-elastic membrane in the lateral dimensions of the experimentally observed spallation layer and with a thickness of 10 µm to infer the approximate magnitude of the elastic constants governing the observed behavior. For the elastic constants, the thickness is a relatively unimportant parameter since the local experimental velocities of the shell enter the simulation as initial conditions, and increasing the thickness does not change the ratio of kinetic energy to elastic energy, both of which depend linearly on the mass and hence volume under the assumption of incompressibility.

The simulations were performed with the finite element solver COMSOL MULTIPHYSICS 6.0 for a linear elastic material with Young’s modulus E, Poisson ratio ν = 0.499 (from E and ν all other elastic moduli follow), density ρ = 1.26 g/cm^3^ and temperature T = 293 K. A Poisson ratio (60) close to 0.5, as we assume, is found for inelastic liquids and also natural rubber. The Poisson ratio is a concept that is strictly applicable only in the case of infinitesimal deformations (61). It is also a dynamic quantity in transient loading conditions and enters nonlinear regimes at high stresses, e.g., in rubbers. Interestingly, our experimental data do not call for more advanced schemes but a constant Poisson ratio. Its value is an input parameter, and we have no direct feedback on whether ν = 0.499 is the best value. However, a different Poisson ratio could affect the determination of Young’s modulus E or the shear modulus by up to a factor of order two since the values for ν are generally limited and any subset of reasonably possible values is even more so.

The problem was solved with a two-dimensional axisymmetric description according to *SI Appendix*, Fig. S7. To increase the stability of the simulation, we interpolated the spallation layer with hexagonal structures, while the outer rim of the spallation disc is fixed in space, representing the bulk of the liquid. Thus, we simulated a film of 160 µm diameter for a laser fluence of 240 ± 11 mJ/cm^2^ (160 µm for 206 ± 9 mJ/cm^2^ and 130 µm for 174 ± 8 mJ/cm^2^). We take the initial condition at t = 0, particularly the velocity profile of different layer segments, from the experimental early plume images (e.g., Fig. 1*C* at 500 ns). The different segments of the shell are assigned the velocity that corresponds to the segment’s lateral position. The segments of the initial film have nine different velocities, see *SI Appendix,* Table S2. While for setting a more “solid-like” modulus E (>1 GPa), we find the mass δm at the membrane tip (Fig. 2*A*) undergoes a harmonic-like motion, for the smaller and relevant rubber-like moduli, we find that the membrane thickness decrease under the stretching of the membrane to values that finally terminate the simulation because the thickness of the film approaches zero and the remeshing of the simulation fails. Under moduli that qualitatively describe the experimental observations, our simulations of a linear-elastic membrane terminate at around $t_{\max}=250\;\mathrm{ns}$ . This is a fraction of the multi-microsecond dynamics we have access to experimentally. Fortunately, for fitting the parameters $h_{\max},\;T$ of a sinusoidal function y=hst=hmax∗sin2πT∗t to observations, only a tiny fraction of the codomain values need to be sampled to obtain accurate results. This is a consequence of a theoretically unbounded domain but bounded codomain and the sine function's favorable low numerical condition number.

For a set of 10 to 15 values of E between 0.1 MPa and 0.2 GPa for each of the three laser fluences with bubble formation, we simulated the plume dynamics up to 0.25 µs after the laser pulse and derived the position vs. time values $h_{s}\left(t\right)$ for the mass point δm (*SI Appendix*, Fig. S7). These values are then fitted by a function of the form $h_{s}\left(t\right)$ with fit parameters $h_{\max},\;T$ . *SI Appendix*, Fig. S8 depicts $T_{\mathrm{sim}}/2$ as a function of E for the three different intensities. From the experimental data, see Fig. 2, we find the fit parameters $T_{\exp},\;h_{\max}$ . To infer the value EsimTexp2 we fitted the simulated data points with a function $\frac{T_{\mathrm{sim}}}{2}\left(E\right)=a*{\left(E-b\right)}^{c}$ , which is a generalized version of the functional relationship between period time and spring constant D of the simple spring pendulum with $T\;\propto \;D^{-0.5}$ but is otherwise just a phenomenological approach that fits the data very well for this purpose. The error bars, often smaller than the size of the datum in the figure, are the statistical fit errors reported from the sinusoidal fit to the simulated $h_{s}\left(t\right)$ data. The values EsimTexp2 of 0.15 ± 0.01, 0.19 ± 0.02, and 0.21 ± 0.01 MPa for the three laser intensities from low to high are found as shown in *SI Appendix*, Fig. S8. The actual error bars of these values are difficult to assess because they can be expected to be dominated by a systematic error rather than relatively small statistical errors.

### Tip Velocity Data.

For a datum to be included in Fig. 2 *B* and *C*, the corresponding plume snapshot must show a well-developed shell with a clearly identifiable tip position. The datum is discarded if a shell has developed too many defects and is significantly compromised, e.g., by the lift-off process (*SI Appendix*, section H). The defects reduce the shell volume available for storing elastic energy from kinetic energy. In extreme cases, this leads to faster overall velocities because the conversion of kinetic to elastic energy is hampered.

### Hole Growth Data.

The plumes develop holes in two different regions and of different quality for analysis. There is a lower region, close to the bulk, where holes develop from early times on, and multiple holes develop simultaneously (Figs. 1*C* and 3*A*). This dynamic is referred to as lift-off (*SI Appendix*, section H) because it can give the impression that the shell will eventually detach from the bulk. The second region is in the upper half of the shell, where often well-separated, relatively isolated growing holes develop, referred to as fracture (Fig. 3 *A*–*C*). For the hole growth analysis, we focus exclusively on these well-separated holes of the fracture (Fig. 3*B*). We find that the fracture holes start as well-defined circular holes and remain approximately circular even at large diameters. Because our plumes are three-dimensional objects and the snapshots are two-dimensional representations, the fractures appear to have noncircular geometry depending on where they develop on the shell and how that position is projected in the image plane (*SI Appendix*, Fig. S9). However, in all snapshots where the projection of the fracture plane happened to be parallel to the image plane, the hole was best described as of circular shape. In order to evaluate all fracture diameters irrespective of position and projection, we always used the greatest distance between two points on the projected rim as the diameter of the fracture in question. This gives a good estimator R of the actual fracture radius $R_{T}$ since it holds $R\leq R_{T}$ with $R<R_{T}$ only for very unfortunate fracture locations with respect to the projection onto the image plane.

Our fracture diameter determination treats them as fractures of a flat two-dimensional sheet and ignores the curvature of the shell. This potentially underestimates the distance traveled by the rim, particularly for larger holes. In addition, the curvature introduces some torque components. We consider the error introduced by this simplification to be of minor influence on the results and immaterial to the conclusions.

While the plume expansion slows down with time and the fracture hole expansions occur on a shell with lower expansion rates, there is still expansion during hole growth. First of all, this expansion is slow compared to the fracture rim velocity of 60 m/s, and second, the main effect of the expansion is to change the topology of the shell on which the rim travels with unchanged velocity. Thus, the hole diameter is still a measure to determine the rim's velocity, and the expansion has a sufficiently small effect on the analysis of the fracture rim speed (*SI Appendix*, section K).

### Elasticity Contribution to the Taylor–Culick Velocity.

Culick, in his work (38), arrives after the integration of Newton’s law $F=\frac{d}{\mathrm{dt}}\left(mv\right)$ and by assuming v=const. at an expression:

[4] $$\sigma \;{r_{0}}^{2}\;\alpha_{0}=\frac{1}{2}\;\rho \;{r_{0}}^{2}\;\delta \;\alpha_{0}\;v^{2}.$$

In polar coordinates r, α with $r_{0}$ the hole radius and $\alpha_{0}$ the azimuthal angle of a polar sector of the hole. The LHS of Eq. **4** is the liberated surface tension energy (of a sector area ${{\frac{1}{2}r}_{0}}^{2}\;\alpha_{0}$ ) of the hole area. The RHS combines energy components of motion. Evers et al. (43) introduced elasticity in the rupture of flat films in an equation similar to Eq. **4** but did so by introducing a positive term on the RHS with elasticity contributing to a reduced TCV because they were concerned about the energy needed for elastic transformations by the traveling rim. Their films were static at the moment of rupture and rested for times $t_{0}$ ≫ $\tau_{i}$ with $\tau_{i}$ being the longest relevant viscoelastic relaxation time.

Without any indication, that a more complex model would add insight to the interpretation of the experimental data, we assume a neo-Hookean elastic behavior (62) for a rubber-like elasticity at large strains. The elastic energy per unit volume is given by Eel=12 G′Λ with $\Lambda \;=\;\lambda_{1}^{2}+\;\lambda_{2}^{2}+\;\lambda_{3}^{2}-3$ , where $G^{\prime }$ is an elastic modulus, here identical to the shear modulus (32), and Λ holds the strain ratios $\lambda_{I}$ for the three axes, most conveniently represented as the z-direction (*SI Appendix*, Fig. S5), along the circumference and in the radial direction as the thickness of the shell, respectively. Introducing $E_{\mathrm{el}}$ as a positive term on the LHS of Eq. **4** and solving for a revised TCV $v_{\mathrm{rev}}$ gives:

[5] $$v_{\mathrm{rev}}=\sqrt{\frac{G^{\prime }\Lambda }{2\rho }+\frac{2\sigma }{\delta \rho }}=\sqrt{\frac{G^{\prime }\Lambda }{2\rho }+v_{0}}.$$

With $v_{0}$ being Culick’s velocity before introducing the elastic term. This is solved for $G^{\prime }$:

[6] $$G^{\prime }=\left({v_{\mathrm{rev}}}^{2}-v_{0}^{2}\right)*\frac{2\rho }{\Lambda }.$$

Which yields with $v^{2}\;\gg \;v_{0}^{2}$:

[7] $$G^{\prime }≅{v_{\mathrm{rev}}}^{2}*\frac{2\rho }{\Lambda }.$$

In order to infer $G^{\prime }$ , knowledge of Λ is mandatory. The inflation dynamics of the observed bubble are adequately described as a biaxial extension (32), in which case it holds:

[8] $$\begin{array}{c} \Lambda ={\lambda_{1}^{2}+\lambda_{2}^{2}+\lambda_{3}^{2}-3=\lambda }_{1}^{2}+\;\lambda_{2}^{2}+\frac{1}{\lambda_{1}^{2}\lambda_{2}^{2}}-3. \end{array}$$

With $\lambda_{3}^{2}=\frac{1}{\lambda_{1}^{2}\lambda_{2}^{2}}$ due to incompressibility. The elongation characteristic along the z-axis of the observed glycerol shell suggests $\lambda_{1}^{2}\gg \lambda_{2}^{2}$ . However, we see no indication that the hole developing in the shell is deviating from rotational symmetry as it should if $\lambda_{1}^{2}\gg \lambda_{2}^{2}$ holds. This suggests that in our dynamic, nonequilibrium situation, rather $\lambda_{1}^{2}\;\approx \;\lambda_{2}^{2}$ is the appropriate assumption in the vicinity of the developing hole. Then with $\lambda_{1}^{2}\;=\;\lambda_{2}^{2}\;=\;\lambda^{2}$ and $\lambda^{2}\gg 1$:

[9] $$\Lambda ={2\lambda }^{2}+\frac{1}{\lambda^{4}}-3≅{2\lambda }^{2}-3,$$

[10] $$G^{\prime }≅{v_{\mathrm{rev}}}^{2}*\frac{\rho }{\lambda^{2}-1.5}.$$

We determined the shell surface area and thickness for bubbles at the 2 μs time point, and together with the condition of constancy of volume and size of the shell base area, that is the shell area around time zero, we inferred $\lambda^{2}=1/\lambda_{3}\approx 11$ . With this value for $\lambda^{2}$ , $G^{\prime }$ becomes an upper limit for two reasons: First, the assumption of equibiaxial stretching minimizes the denominator in Eq. **10**. Second, we assumed a location-independent shell thickness to reach $\lambda^{2}=11$ , which we know is not categorically true since the shell typically becomes thinner toward the tip and the local strain ratios are eventually larger than its average.

For an equibiaxial extension, $\lambda_{1}^{2}=\lambda_{2}^{2}=\lambda^{2}$ , the stress is equal in all directions in-plane. In the case of a thin shell with an initial thickness $\delta_{0}$ this stress can be interpreted as a kind of surface tension, that is, a force $F_{\sigma }$ (32) per unit length, practically independent of the strain for larger strains.

[11] $$F_{\sigma }=G^{\prime }\delta_{0}\left(1-\lambda^{-6}\right).$$

### Elastic Energy vs. Surface Energy.

We have simulated energy distribution in the moving shell using COMSOL MULTIPHYSICS 6.0, as described above. The relevant energies for the overall balance are the kinetic energy $E_{v}\left(t\right)$ , elastic energy $E_{G}\left(t\right)$ , and surface energy $E_{\sigma }\left(t\right)$ . $E_{G}\left(t\right)$ is proportional to the volume of the shell, while the surface energy is proportional to the area of the shell. For a hypothetical shell with neo-Hookean behavior and Young’s modulus of 170 MPa we can simulate the dynamic up to the time point where the kinetic energy is zero and converted into elastic energy. The dynamic of the mass point δm is again sinusoidal in analogy to our experimental data of Fig. 2, where Young’s modulus was found to be much smaller. *SI Appendix*, Fig. S10 depicts the temporal evolution of $E_{G}$ up to a point around t′ = 250 ns where $E_{G}$ is maximal and normalized to one with $E_{v}=0$ . If surface energy would be the dominant sink of kinetic energy in any scenario, then $E_{\sigma }\left(t^{\prime }\right)=1$ is required instead. However, comparing these two hypothetical scenarios, see *SI Appendix*, Fig. S10, it is evident that $E_{G}\left(t\right)\neq E_{\sigma }\left(t\right)$ holds for most t. Nevertheless, if $E_{G}\left(t\right)$ leads to a sinusoidal behavior for the dynamic of the mass point δm, a scenario with surface energy as the dominant sink cannot lead to the same well-fitted sinusoidal behavior found in the experimental data of Fig. 2.

## Supplementary Material

Appendix 01 (PDF)

Movie S1. Temporal evolution of the laser-induced bubble in glycerol. The bubble’s temporal evolution for the first 3 μs under vacuum conditions of 10^−3^ mbar, initiated by an ablation fluence of 240±11 mJ/cm^2^. The movie-like series of snapshots is not the temporal evolution of a single bubble but the temporally ordered sequence of individual snapshots of different ablation events recorded at different time points spanning over 3 μs.

## Data Availability

Image raw data, Computer code data have been deposited in https://edmond.mpdl.mpg.de (10.17617/3.BPNGXA) (63). Some study data available. (We have used the commercially available software COMSOL MULTIPHYSICS 6.0 for creating simulation data. The code is not available. The software is available from: Comsol Multiphysics GmbH, Robert-Gernhardt-Platz 1, 37073 Göttingen, Germany).
