# Supplementary material for: Rubber-like elasticity in laser-driven free surface flow of a Newtonian fluid
Source: Proc Natl Acad Sci U S A. 2023 Jun 26;120(27):e2301956120. doi: 10.1073/pnas.2301956120 (PMC10319024; doi:10.1073/pnas.2301956120)
Supplement: Supplementary file 1 — Appendix 01 (PDF) [file pnas.2301956120.sapp.pdf]

PNAS

Supporting Information for

Rubber-like elasticity in laser-driven free surface flow of a Newtonian fluid.

Meghanad Kayanattil, Zhipeng Huang, Djordje Gitaric, Sascha W. Epp*

*To whom correspondence may be addressed: Sascha W. Epp

Email: sascha.epp@mpsd.mpg.de

This PDF file includes:

Supporting text
Figures S1 to S15
Tables S1 to S3
Legend for Movie S1
SI References

Other supporting materials for this manuscript include the following:

Movie S1

Supporting Information Text

A. Light absorption in water and glycerol

Water and glycerol have strong light absorption in the infrared spectral range around 3 µm wavelength (3400 cm^{-1} wavenumber), see Fig. S11. The absorption coefficient at 2.94 µm wavelength and with our experimental ablation fluences is approximately 8300 cm^{-1} for water (1). For glycerol, fluence-dependent absorption coefficient information is lacking in the existing literature and the best available value is 6500 cm^{-1} (2). The strong absorption is based on resonant excitation of O-H bond stretching modes prevalent in both compounds. Due to the strong interaction between the molecules by means of hydrogen bonding, the excitation energy redistributes quickly over the entire ensemble. For a comprehensive absorption spectrum of water from 40 nm to 1 mm wavelength, see Fig. 1 of ref. (3).

Details on the interaction between a sub-nanosecond laser and water at high fluences in ambient conditions can be found in ref. (4). The fluences used for such ablations are five times higher than those necessary for bubble formation. A higher fluence results predominantly in phase explosion instead of photomechanical spallation (see Sec. B).
Similarly, numerous studies using mid-IR nanosecond lasers have tackled the laser ablation of liquids under ambient conditions, particularly for water or water-rich biological targets such as tissues (5–7). Cheng et al. (5, 6) employed a 1-D fluid dynamics model to simulate plume dynamics after ablation, building on the experimental work of Aptiz and Vogel (8), who demonstrated ablation with a 70 ns, 2.94 µm laser. However, these studies also primarily concentrated on the phase explosion process, which dominates at higher fluences, and not the photomechanical spallation, where we observe laser-driven bubbles.

B. Photomechanical ablation efficiency and local temperature increase

Photomechanical effects can alter the efficiency of material ejection (9, 10) during general laser ablation events. In a pure photomechanical ablation process, a thin layer near the liquid-vacuum interface is heated by a short laser pulse to temperatures insufficient for vaporization but at such a high rate that it cannot undergo thermal expansion. This creates a traveling compressive stress wave. Due to a notable difference in acoustic impedance between the liquid and vacuum, the wave reflects at the free surface. As a consequence, a tensile component can form and grow in amplitude with increasing depth. If this tensile wave amplitude surpasses the material’s tensile strength, a thin layer can be detached (11, 12). A schematic description of such an event is available in Fig. S12. The efficiency of photomechanical ablation depends on the material's properties, especially its tensile strength, absorption length, and the Grüneisen parameter. The Grüneisen parameter determines the initial shock pressure being induced in the system (11, 12).

The occurrence of photomechanical effects can increase the ejection efficiency of ablation, which means that of the available pulse energy, less is converted into heat resulting in a reduced temperature in the target. We are unaware of studies where the local temperature increase was probed in detail in the case of pulsed laser ablation of liquids. The presumably transient nature of the associated material parameters during laser interaction and afterward will make the problem particularly challenging to tackle. There is little consensus regarding the Grüneisen parameter and the dynamic tensile strength, especially for glycerol, at these extreme conditions.

Thus, we will perform a best-effort estimation of the maximum shell temperatures in the system. We can deduce a qualitative understanding of the temperature increase during the stress-confined ablation utilizing the isochoric heat capacity C_v. While this parameter is not at our disposal for glycerol at the relevant pressure ranges (>100 MPa), we rely on recent work by Ahmadi et al. (13) at a pressure of 55 Mpa, where $C_v \approx 2142$ J/(Kg·K) is reported for a 298 K isotherm. With knowledge of the volume of material interacting with the laser pulse, the temperature increase can be calculated. The interaction volume is given by the area over which a bubble is formed times the

depth where 90 percent of the laser energy is absorbed. For an absorption coefficient of $\mu_a = 6500\ \mathrm{cm}^{-1}$, 90% of the laser energy will be absorbed within 3.5 µm. For this cylindrical volume of 3.5 µm height and radius of 80 µm, 43 µJ will be deposited by the laser pulse with a peak fluence of 240±11 mJ/cm^2. Assuming all 90% of the laser energy is converted to heat, we get an upper estimate of the average local temperature increase of about 200 K resulting in an absolute temperature of about 500 K. This is well below the boiling point of glycerol (≈ 560 K) (14) and suggests a liquid shell, as supported by our observations. However, due to the exponential absorption of laser energy, layers up to a depth of less than 1.5 µm could be heated above the boiling point in this limiting scenario.

C. One-Dimensional solution of the thermoelastic wave equation in a liquid and minimum spall fracture depth for glycerol calculation

A detailed discussion regarding the photomechanical spallation process can be found in the article by Dingus and Scammon (11). The important takeaway for us from this work is the qualitative prediction they have concerning the spallation depth. They estimate the spallation to occur at a depth one or a few times the absorption length of the ablation laser. In our case, the absorption length is around 1.5 µm (for $\mu_a = 6500\ \mathrm{cm}^{-1}$), giving a rough spallation depth of a few micrometers.

To better understand the process, we used a simple model describing the photomechanical responses in liquids after laser pulse absorption, which follows ref. (15). Using this model, a qualitative understanding of the transient stresses induced during our ablations can be gained. To this extent, we follow a slightly modified process described by Paltauf et al. (12) for top-hat pulses (spatial domain) to estimate the thickness range of the spall layer in our pico-second laser-induced ablations.

In the case of stress-confined ablations, when for the pulse width t_p and the acoustic dissipation time t_{ac} holds $t_p << t_{ac}$, the absorption of the top-hat laser pulse produces an initial pressure distribution $p_0(d)$:

$$p_0(d) = \mu_a \Gamma H_0 \exp(\mu_a * d) \tag{S1}$$

Where Γ is the Grüneisen parameter of the liquid, relating the initial pressure to the absorbed volumetric energy, H_0 is the fluence of the laser, μ_a is the liquid's absorption coefficient for the ablation laser's specific wavelength, and d is the depth in the sample. In the case of liquid glycerol at room temperature, we find $\Gamma = 0.9$ (16) and $\mu_a = 6500\ \mathrm{cm}^{-1}$ (2).

Following the absorption, compressive stress waves are generated. These generated waves consist of two parts with the same spatial profile as p_0, but half the amplitude, traveling in opposite directions. The part propagating towards the free surface is reflected at the surface with a reflection coefficient $R_{ac} = \frac{Z_2 - Z_1}{Z_2 + Z_1}$. Z_1 and Z_2 are the acoustic impedances of the liquid (for glycerol 2.4*10^6 kg/m^{2}s (17)) and the surrounding medium, respectively. In our case, the surrounding medium is a vacuum. Thus, irrespective of the acoustic impedance of the sample, we have the reflection coefficient $R_{ac} = -1$. Then the amplitude $p_\delta(d,t)$ of the stress wave at a depth d > 0 can be written as a sum of 3 pressure components p_1, p_2, p_3,

$$p_\delta(d,t) = p_1 + p_2 + p_3 \tag{S2}$$

$$p_1 = \begin{cases} 0.5 p_{0,\max} \exp\left(-\mu_a (d - c_s t)\right), & d > c_s t \\ 0, & \text{otherwise} \end{cases} \tag{S3}$$

$$p_2 = \begin{cases} 0.5 p_{0,\max} \exp\left(-\mu_a (d + c_s t)\right), & d > 0 \\ 0, & \text{otherwise} \end{cases} \tag{S4}$$

$$p_3 = \begin{cases} 0.5 p_{0,\max} R_{ac} \exp\left(-\mu_a (c_s t - d)\right), & c_s t > d > 0 \\ 0, & \text{otherwise} \end{cases} \tag{S5}$$

Where c_s is the speed of sound in the medium for glycerol $c_s \approx 1890\,\mathrm{m/s}$, and $p_{0,\max} = \mu_a \Gamma H_0$.

The influence of a finite laser pulse duration can be incorporated by convolution with the temporal profile of the laser for a revised pressure amplitude as a function of time and depth:

$$p(d,t) = g(t) \otimes p_\delta(d,t) \tag{S6}$$

Our modeling uses $g(t)$ as a Gaussian temporal distribution of sigma ≈ 170 pico-seconds (corresponding to a FWHM of 400 ps) centered at around 600 pico-seconds. We do not know when the spallation is happening as this is dependent on transient parameters of the liquid outside of these equations, like a temperature-dependent spallation strength. Hence, the modeling is carried out for a broad temporal window of 8ns (Fig. S13A).

We calculated the time-dependent hypothetical spall depth (HSD) (Fig. S13B), which is simply the depth at which the pressure amplitude reaches the dynamic tensile strength (18) of glycerol (85 MPa at 293 K). At these given conditions, HSD can reach a depth of up to 12 µm within 8 ns. The lowest depth at which a potential spall fracture could happen is around 300 nm very early after absorption. Given the temperature-dependent variations of Γ and fluence-dependent variations in μ_a these values may vary significantly. However, a clear behavior concerning the spatial fluence distribution and its effect on the shell thickness can be garnered from the calculations. In this model, lower pulse energies lead to lower fluences for the top hat pulse and an increased HSD (Fig. S13B). In our experiment, we observed higher shell thickness for the parts closer to the bulk and thinner shells for the top features of the plume. This observation can be attributed to the Gaussian spatial intensity distribution of our ablation pulse, where the $p_{0,max}$ is not constant in the spatial domain. The top-hat pulse used in modeling fails to account for this experiment's boundary conditions. With the HSD calculations available for different pulse energies (Fig. S13B), we can infer that the wings of the Gaussian spatial intensity distribution with lower fluence likely result in higher HSD and thicker shells.

D. Relation of the observed dynamics to laser-induced forward transfer (LIFT)

The LIFT process (19) is a laser-driven material ejection process where also bubble formation is observed (20, 21). However, comparing the LIFT dynamic with our process more rigorously, the similarities seem superficial. The mechanism of material ejection due to photomechanical stress waves differs from the phase explosion induced material ejection in LIFT. In the latter, the material is driven by an expanding vaporized volume, while photomechanical spallation happens ideally under the absence of vaporization, and only a minimal amount of energy contributes to phase change, apart from some evaporation within voids and cracks (12).

Between LIFT and photomechanical spallation exist considerable differences in typical values of parameters and observables regarding laser wavelength, pulse length, and laser energy. Furthermore, there are differences in the geometry of the ablation process and the induced physical processes. Typically, the LIFT process is performed in a back-illumination geometry, where a highly focused laser enters the backside of a target and creates an ablation of the front side of the target, see Fig. 5 of (19). The ablation of the front is initially driven by a pressurized volume of vaporized target created in some depth below the front surface.

Our experimental setup (SI Appendix, Fig. S6) is realized in front illumination, where the target is hit from the front, and the front section is ablated. The ablated material propagates in the direction from which the laser came. This geometrical difference implies that the ablated material has

interacted with the highest intensities of the laser. At LIFT, the ejected material is from a region that did not experience the high fluences present in the vaporization region.

The lasers employed in LIFT, particularly in experiments (20) with liquid targets such as glycerol, typically have nanosecond pulse lengths and wavelengths in the shorter part of the visible spectrum, e.g., around 350 nm and below. If we consider the absorption spectrum of water, which we find sufficiently similar also to the one of glycerol, we find the region around 350 nm to be the one with the longest absorption length and hence weakest absorption. In fact, the difference in absorption at 350 nm and 3000 nm, where we operate in our setup, is eight orders of magnitude (see Fig. 1 of ref. (3)). In our experiment, the matter with the most intense interaction with the laser is the most relevant for the observable dynamics. In LIFT, the matter with the least interaction with the laser light forms the matter of the early plume dynamic.

In LIFT, the laser is highly focused, allowing for fluences that are 100-1000 times larger than in our case (20, 21). As a result, the pressure generated during this phase explosion driven bubble expansion is generally one order of magnitude higher than that due to the mere thermoelastic effect (22). These differences in laser parameters, energy absorption, and geometry lead to considerable differences in the observable dynamics. Our dynamics are pretty much covered by a few microseconds, while in LIFT, the dynamics last several dozens of microseconds and hence ten times, or more, longer.

The LIFT process is typically carried out under atmospheric conditions. Our process is not realizable if not carried out in a vacuum environment. The shell is only formed if not subjected to any backpressure. At atmospheric pressure, the environmental air molecules immediately destroy the shell due to the creation of many nucleation sites from air-liquid interaction.

E. Kelvin-Voigt and Maxwell rheological models

Rheological models are frequently visualized by a network of springs, resembling the elastic contribution, where the force is linearly dependent on the elongation following Hooke's law, and dashpots, resembling the dissipative viscous contribution, where a Newtonian fluid is assumed and the force is proportional to the temporal change of elongation.

The simplest manifestation of the Maxwell model (23) contains two hypothetical elements, a single dashpot in series with a single spring. In such a series network, the stress in the dashpot is always identical to the stress in the spring. The total strain γ, however, is always the sum of the individual strain in each element. If this combination is suddenly deformed, then the stress decays on a characteristic timescale τ, the relaxation time given by the viscosity η of the dashpot divided by the elastic constant E of the spring. It shows the viscous flow for a timescale much longer than the relaxation time and elastic resistance as an initial response to fast strains. The constitutive equation is given by:

$$\sigma = \eta \frac{\partial \gamma}{\partial t} - \tau \frac{\partial \sigma}{\partial t} \tag{S7}$$

The generalized Maxwell model is a more complex network of parallel models. In particular, a single spring is parallel to a number of n Maxwell models (each with spring and dashpot in series) like Eq. S7 and generally different viscosity, elasticity, and hence relaxation times. Due to the single spring in parallel, it can have elasticity for infinite times.

The Kelvin-Voigt model (23) is as simple as the Maxwell model and is given by a parallel network of spring and dashpot, respectively. In the parallel network, the strains, not the stresses, are identical in each branch and identical to the total strain. The total stress is the sum of the individual stresses in each branch. We find a constitutive equation of the form:

$$\sigma = E\,\gamma + \eta\frac{\partial\gamma}{\partial t} \tag{S8}$$

Suppose we would add a mass point δm to the otherwise mass-less network and solve for its motion Eq. S8 can be transferred to the standard form of the harmonic oscillator:

$$\frac{\partial^2\gamma}{\partial t^2} + 2\xi\omega_0\frac{\partial\gamma}{\partial t} + \omega_0^2\,\gamma = 0 \tag{S9}$$

with new parameters $\xi$and ω_0 representing a dimensionless damping ratio and the frequency of the periodic motion, respectively. These parameters are, of course, functions of parameters of Eq. S8 and δm. The well-known solutions of Eq. S9 with amplitude A and phase φ are:

$$\gamma(t) = Ae^{-\xi w_0 t}\sin(\sqrt{1-\xi^2}\omega_0 t + \varphi) \tag{S10}$$

The velocity (and η) dependent term $2\xi\omega_0\dot{\gamma}$ of Eq. S9 leads to a sinusoidal motion with damped amplitude and altered frequency.

The Kelvin-Voigt model shows instantaneous viscous behavior since the straining of the spring and the dashpot, which must work for every deformation, co-occurs. The Kelvin-Voigt model is a simple model for solids with viscosity and less meant for fluids. For a constant force, the deformation is damped by viscosity and the system will finally reach a state of pure elasticity. The Kelvin-Voigt model is an entry point for describing an elastic solid rather than a liquid subject to viscous energy dissipation.

While the Maxwell element constitutes a high-pass with low attenuation for fast dynamics, the Kelvin-Voigt element resembles a low-pass with high attenuation for fast dynamics.

We simulated the behavior of the membrane under the assumption of a Maxwell rheological model. For that, we kept the spring's elastic constant, here in the form of the shear modulus G', constant and used different parameters for the viscosity and relaxation time. We used $G' = 3 * 10^6$Pa, which is about ten times larger than our experimentally found elastic constant and reduced the membrane's kinetic energy by going to a tenth of the velocity. This ensured we could reach the quarter period point within the simulation, at which we have maximum elongation. The challenge lies in the significant shape changes of the simulation domain.

Fig. S14A depicts the position of the mass point δm as a function of time, similar to Fig. 2B and C. We find that for different viscosities and hence relaxation times according to $\eta = \tau_M * G'$ the motion of the mass point δm follows a sine-like behavior at least for the time till the maximum position is reached. This is true for the fully elastic membrane but also allows Maxwell models with different viscosities η to produce a motion of δm, that is potentially identifiable with a sine-like appearance, given the present experimental resolution. What these Maxwell models separate from an actual harmonic behavior becomes evident if we inspect the distribution of the total energy to the branches of kinetic and elastic energy. Fig. S14B shows exemplarily the distribution of energies for the simulation with $\eta = 1.4$ Pa*s. While in an actual harmonic motion, the energy at maximum elongation is entirely converted from kinetic energy to elastic potential energy, for the Maxwell model with $\eta = 1.4$ Pa*s, the energy is not stored but got dissipated and, as such, is not available for any dynamic, like a hole opening dynamic at high velocity or a jetting of matter. Such a situation can be experienced in the slow blowing of chewing gum bubbles (24). Once the driving pressure is removed, the shell is dynamic-less and just subject to gravity.

F. Dimensionless flow numbers

Surface flows can be categorized by dimensionless numbers (25) expressing the relative influence of different forces such as capillarity (surface tension), viscosity, inertia, or gravity. The capillary number (Ca) expresses the relative importance of viscous drag forces over capillary forces. High Ca indicates the dominance of viscous forces over capillary forces. The capillary number is defined by the viscosity η, a characteristic velocity v and the surface tension σ:

$$Ca = \frac{\eta * v}{\sigma} \tag{S11}$$

With the parameters of glycerol and the experimental conditions of $\eta = 1.4$ Pa s, $v = 270$ m/s, and $\sigma = 0.063$ N/m we find $C_{\mathrm{PL}} \equiv Ca \approx 6000$.

The Reynolds number R relates the influence of inertial forces over viscous forces. At low R, viscous forces dominate. It is the regime of laminar flow with smooth fluid motion. At high R, inertial forces dominate and the flow is turbulent. The Reynolds number is defined by the density ρ, viscosity η, a characteristic velocity v, and length scale r_B:

$$R = \frac{\rho * r_B * v}{\eta} \tag{S12}$$

With $\rho = 1260$ kg/m^3 and a characteristic length identified as the bubble radius $r_B = 80$ µm we find $R_{\mathrm{PL}} \equiv R \approx 18$.

An elasto-capillary number E_c suitable for the present experimental conditions can be defined by following ref. (24) and utilizing the shear modulus G:

$$E_c = \frac{r_B * G}{\sigma} \tag{S13}$$

E_c relates elastic forces with capillary forces expressed by the surface tension. r_B expresses again typical dimensions of the flow and is used in analogy to the radius in a spherical shell.

G. Sine-like tip motion for shells under the Maxwell model

Not only fully elastic membranes can exhibit a sine-like motion for the tip, but also shells assumed to be of a material following the Maxwell model might. Under the Maxwell model (SI Appendix, Sec. E), the material has an elastic constant G' and a viscosity η. From these two parameters follows directly a relaxation time $\tau = \eta/G'$. Fig. S14A shows simulation results of the tip position for different sets of parameters G', η. The results of the different simulations can be reasonably fitted by simple sine functions. The difference to the sine motion in a pure elastic shell case is in the temporal development of the kinetic and elastic energy in the shell. Fig. S14B shows that for the Maxwell model, the kinetic and the elastic branch of energy in the shell can be (entirely) depleted in the sine's quarter period, leaving no energy in the shell to perform any dynamic past the quarter period point. For the fully elastic shell all initial kinetic energy would be readily available in the form of elastic energy at the quarter period point, e.g., for a fast hole opening dynamic.

H. Lift-off

The lift-off is a defect of the shell in the lower third part, close to the bulk, where the prevalence to multiple nucleation and rupture reduces the area of the shell that is connected to the bulk. Finally, this can largely detach the shell from the bulk.

Our work shows that higher straining rates coincide with higher elasticity, stabilizing the shell. The region where the lift-off occurs has higher shell thickness and lower strain rates than other areas.

Both findings are adverse for a strong elasticity and a stabilization of the shell. Additionally, the droplets traveling in the volume encompassed by the shell are potential sources of rupture once they get in contact with the shell. While this contact seems unlikely to happen in the upper region of the shell due to the lower velocity of the droplets in comparison to the shell, it seems likely in the region where the rupture for the lift-off takes place.
The multiple rupture sites in the lift-off region, where holes also dynamically combine to form larger holes prevent us from performing a statistical analysis to determine the hole growth velocity and infer elastic parameters. We cannot make a comparison to the holes in the upper section and get information for different sections of the shell.

I. Storage modulus for liquid glycerol at different frequencies

The idea that there might be a measurable storage modulus (G') in Newtonian liquids well above the molecular relaxation time scales is nothing new (26). In Table S3, we present the studies which have looked at the storage modulus G' of glycerol at different frequencies. G' is measured under infinitesimal strain and only indirectly related to the elasticity at large strains.
Typically, measurements of the storage modulus are dynamic measurements of small-amplitude oscillations as a function of frequency $G' = G'(\omega)$ with the limiting immediate response value $G'_0 = G'(\infty)$. From our measurement we determine an elastic constant $G = G(t)$ as function of time. These are different quantities and both are measures of stored elastic energy. For the limiting case presumably holds $G'(\infty) = G(0)$ (27). However, the common relation between a frequency ω and a transient t of the form $\omega = {}^{1}/_{t}$ does not hold generally in the relation for the storage modulus and we have $G'(\omega) \neq G(1/\omega)$ outside the limiting case (27). Nevertheless, since Table S3 lists storage moduli as a function of frequency, it might still be illustrative to take our estimated relaxation time of 10 µs and the derived elastic constant $G_{\mathrm{exp}} = 0.48\ \mathrm{MPa}$ to be a frequency dependent modulus $G'(\omega) = G_{\mathrm{exp}}\left(\frac{1}{10^{-5}\mathrm{s}}\right) = G_{\mathrm{exp}}(0.1\ \mathrm{MHz})$

J. Traveling rim

The rim's origin, shape, and velocity under varying influence of viscous, inertia, or surface forces draws steady attention (28–36). For circular hole growth, we can follow ref. (32), where the authors differentiate three regimes depending on the Ohnesorge number $Oh = \eta/\sqrt{2\rho\sigma\delta}$ relating viscosity to inertia (density ρ, film thickness δ) and surface tension σ. For high $Oh > 10$ it is found that the toroidal rim is not forming; instead, the whole film thickness is increasing over a certain region. Under our experimental conditions, the Ohnesorge number is $Oh_{\mathrm{exp}} \approx 3$. If we assume a 10-fold increased viscosity, the Ohnesorge number increases by the same factor and we would enter a parameter region where we can expect the influence of viscosity on the shape of the rim and the velocity with which the rim travels. An exponential velocity growth over time towards the Taylor-Culick velocity should develop, which is not observed.
However, caution is advised in this conclusion. We have shown that the shear elasticity can disguise as apparent surface tension. This apparent surface tension can be calculated by solving the expression of the Taylor-Culick velocity for the surface tension:

$$\sigma_{\mathrm{ap}} = \frac{v^2 * \rho * \delta}{2} \tag{S14}$$

With our parameters, we find an apparent surface tension about 320 times larger than the bulk surface tension. The Ohnesorge number would decrease by almost a factor of 20; hence, the dynamic viscosity could increase by another factor of 20 while still having Ohnesorge numbers of 10 and below. Hence, a much more significant dynamic viscosity increase during the plume dynamic could be possible, although the rim shape suggests conditions of an inviscid liquid.

K. Holes fracture

The hole fracture, which we analyzed to infer the rim speed, happens in the upper half of the shell and generally at times when the expansion of the shell has already slowed down. Nevertheless, an underlying expansion is potentially present during the hole growth dynamic. In the analysis of the rim speed, we neglect any influence of this underlying motion on the rim speed.

Fig. S15 depicts a small section of the expanding shell and the velocity components near the onset of a hole fracture. The cyan disc indicates the hole opening. The mass points in close vicinity to the rupture have different velocity components. A velocity component indicated by the red arrows reflects the outward motion of inflating volume and is parallel to the surface normal and perpendicular to the rim motion. Then there is a velocity component indicated in green that reflects that the surface is increasing. For an observer in the point of rupture, all other points on the surface move away and increase distance. This effect is entirely independent of any traveling rim. The red velocity field, perpendicular to the motion of the traveling rim, overlays with the rim motion without interference and effect on the magnitude of the rim motion in the surface plane. The green velocity components are small in magnitude compared to the rim speed, which is 60 µm/µs, since the green motion is the relative motion of neighboring points. The maximal velocity difference between any two points on the entire shell, at any time of the dynamic, is only a fraction of the tip's maximal 300 µm/µs velocity since the shell is a flat disc in the beginning that bulges. Hence, the rim’s traveling speed is very high compared to local differences in the shell's velocity, even for well-separated points on the shell. That is why the holes finally “eat up” the entire shell.

Furthermore, the green velocity components can be understood in terms of how they increase the stress in the shell because the stress is finally responsible for the speed of the rim. We have shown in the report that the elasticity in the shell can be expressed as an apparent surface tension, as expressed in Eq. 11 in the Materials & Methods section. At some point of inflation, this surface tension becomes independent from further extension due to power scaling in the formula. Consequently, the forces do not increase, although the extension increases. So, even if the velocity components do increase the general extension of the shell, the influence on the forces is small and the velocity might only be influenced by the fact that a stretched and hence thinner shell has a rim with less mass at a given hole diameter. This change in thickness is again a slow process compared to the dynamic of a traveling rim.

In total, the effect of the velocity components of the shell is mainly that they dynamically affect the topology the rim is traveling on, but not relevantly the velocity with which the rim travels on the surface of the shell. The underlying expansion changes the time it takes for the rim to reach a point on the shell at a certain distance to the fracture point but not the velocity with which it travels the potentially larger distance toward this point.

L. Elasticity in charged liquids

Charging and potentially related strong electric fields have been identified in static experiments to promote elastic properties (37). Short pulse laser-matter interaction can potentially charge the liquid via plasma creation. However, the use of 3 µm wavelength radiation greatly obfuscates the required multi-photon interaction. We do not detect light emission accompanying any plasma creation at the employed laser intensities, although detectable in our setup at considerably higher energies.

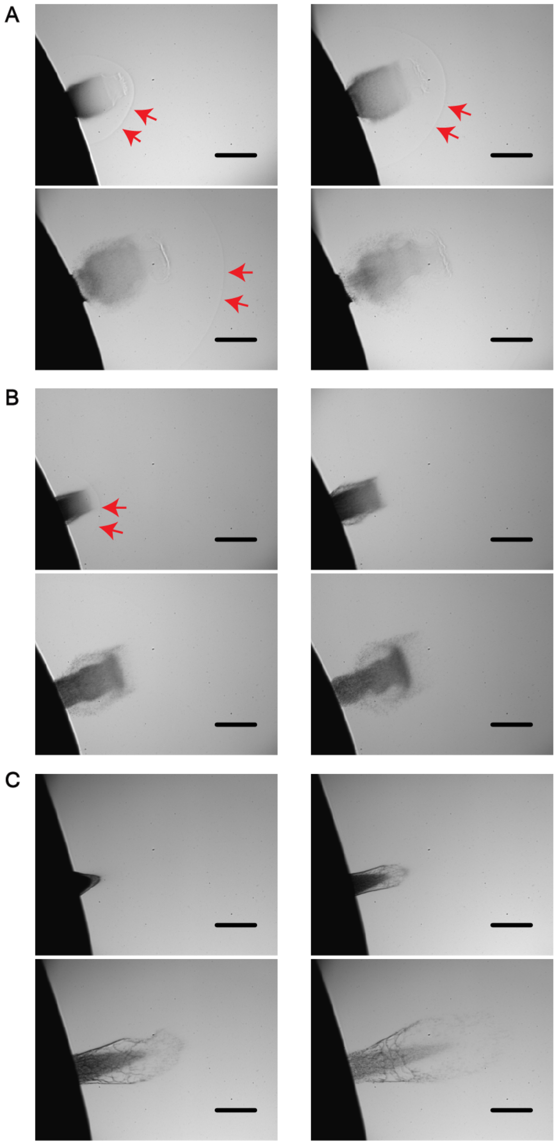

Fig. S1. High fluence ablation of water and glycerol under ambient conditions (atmospheric pressure). (**A**) Temporal progression of the plumes for ambient ablation of water for an ablation fluence of 550±33 mJ/cm^2. The top row, left to right, is 500 ns, 1 µs, and the bottom row is 2 µs and 3 µs. The red arrows indicate the shockwave front. The black scale bar corresponds to 300 µm. (**B**) Temporal progression of the plumes for ambient ablation of glycerol for an ablation fluence of 550±33 mJ/cm^2. The top row, left to right, is 500 ns, 1 µs, and the bottom row is 2 µs and 3 µs. The red arrows indicate the shockwave front. The black scale bar corresponds to 300 µm. (**C**) Temporal progression of the plumes for vacuum ablation of glycerol for an ablation fluence of 455±20 mJ/cm^2. The top row, left to right, is 500 ns, 1 µs, and the bottom row is 2 µs and 3 µs. The black scale bar corresponds to 300 µm.

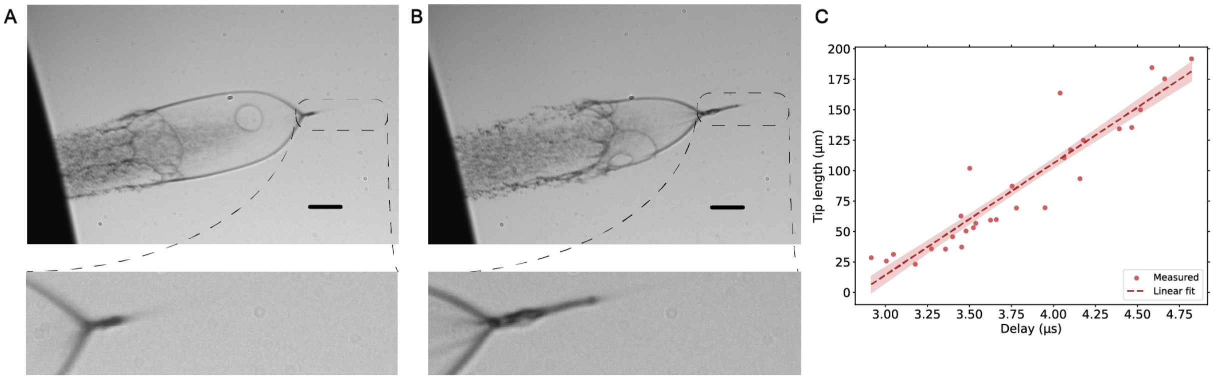

Fig. S2. Bubble lift-off and tip jetting for high laser peak fluence. (**A**) Bubble snapshot at a delay of 3.6 µs where the lift-off from bulk developed and the tip starts jetting (tip length of around 60 µm) along with zoomed-in image as indicated. The black scale bar corresponds to 100 µm. (**B**) Similar to (**A**) but at a delay of 4.4 µs where the bubble largely disintegrated and the tip jetting reaches a length of about 130 µm. The black scale bar corresponds to 100 µm. (**C**) Tip jet length vs. delay plotted along with the linear fit for the data giving a velocity of 92±7 m/s. The shaded area represents the 68% confidence band of the fit.

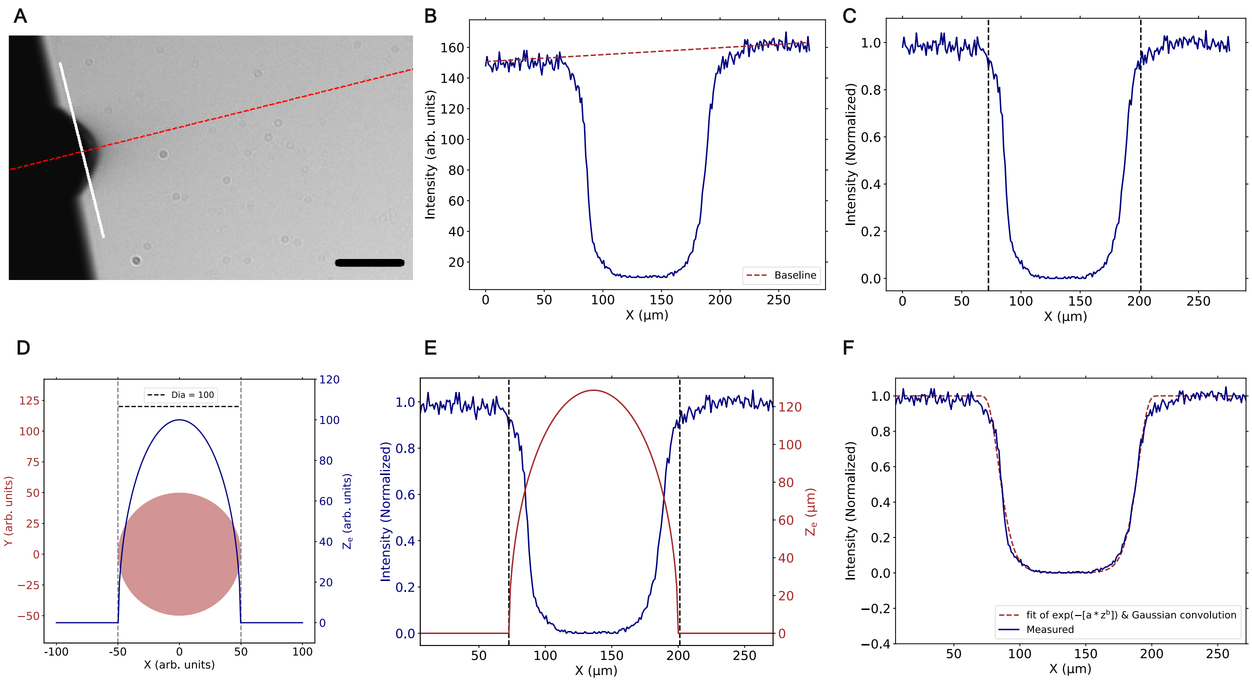

Fig. S3. Transfer function parameter determination from early spall intensity profiles. (**A**) An early spall at about 190 ns for an ablation fluence of 240±11 mJ/cm^2. The white line indicates the intensity profile location. The dashed red line lies along the z-axis. The black scale bar corresponds to 100 µm. (**B**) Raw intensity profile (blue) and a skewed baseline (dashed, brown). (**C**) Normalized and baseline-corrected intensity profile (blue). (**D**) Geometrical path length (blue, arb. units) as a function of the lateral position of a disc or cylindrical object (light brown) with a rotational axis perpendicular to the paper plane. (**E**) Normalized intensity profile (blue) from (**C**) with initial size guess of a geometrical object as indicated in (**D**). (**F**) Normalized intensity profile (blue) from (**C**) and fit to this profile based on the transfer function $exp\left(-aZ_e{}^b\right)$ with Z_e derived from optimization of the size of the cylindrical object, see panel (**E**). The fit also incorporates a convolution of a Gaussian point spread function to account for the imaging setup's limited optical resolution (dashed, brown).

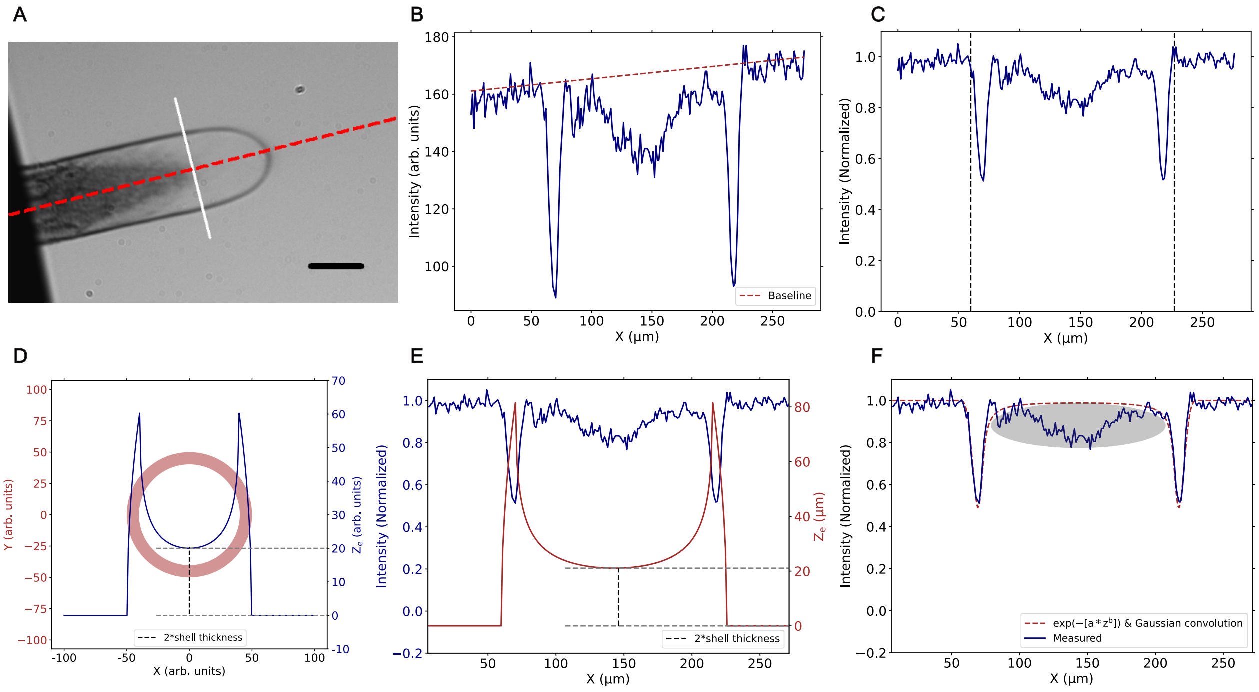

Fig. S4. Bubble shell thickness estimation**.** (**A**) A bubble at delay 1.8 µs for an ablation fluence of 240±11 mJ/cm^2. The white line indicates the intensity profile location. The dashed red line lies along the z-axis. The black scale bar corresponds to 100 µm. (**B**) Raw intensity profile (blue) and skewed baseline (dashed, brown). (**C**) Normalized and baseline-corrected intensity profile (blue). (**D**) Geometrical path length (blue, arb. units) as a function of the lateral position of a ring or hollow-cylindrical object in projection (light brown) with a rotational axis perpendicular to the paper plane. (**E**) Normalized intensity profile (blue) from (**C**) with initial size guess of a geometrical object as indicated in (**D**). (**F**) Normalized intensity profile (blue) and the transfer function $exp\left(-aZ_e{}^b\right)$ with parameters determined from Fig. S3. and Z_e (with Z_e derived from optimization of the shell thickness of the hollow-cylindrical object, see panel (**E**). The fit also incorporates a convolution of a Gaussian point spread function to account for the imaging setup's limited optical resolution (dashed, brown). Gray-shaded data are excluded from the procedure.

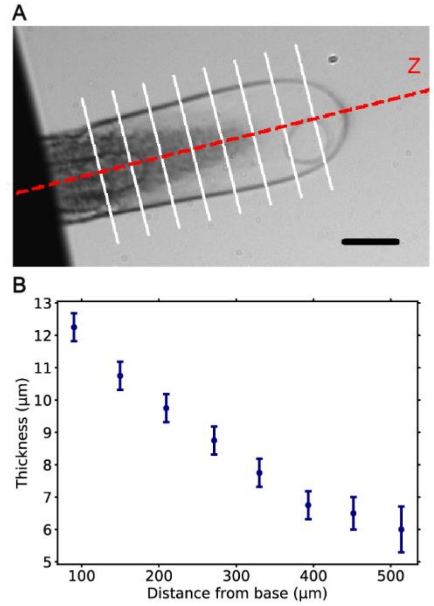

Fig. S5. Bubble shell thickness along the z-axis. (**A**) A bubble was generated with an ablation fluence of 240±11 mJ/cm^2 at a delay of about 2 µs. White lines perpendicular to the z-axis (dashed, red) indicate the locations where the thickness of the shell was extracted by fitting the intensity profile. The black scale bar corresponds to 100 µm. (**B**) Shell thickness at different positions, see panel (**A**).

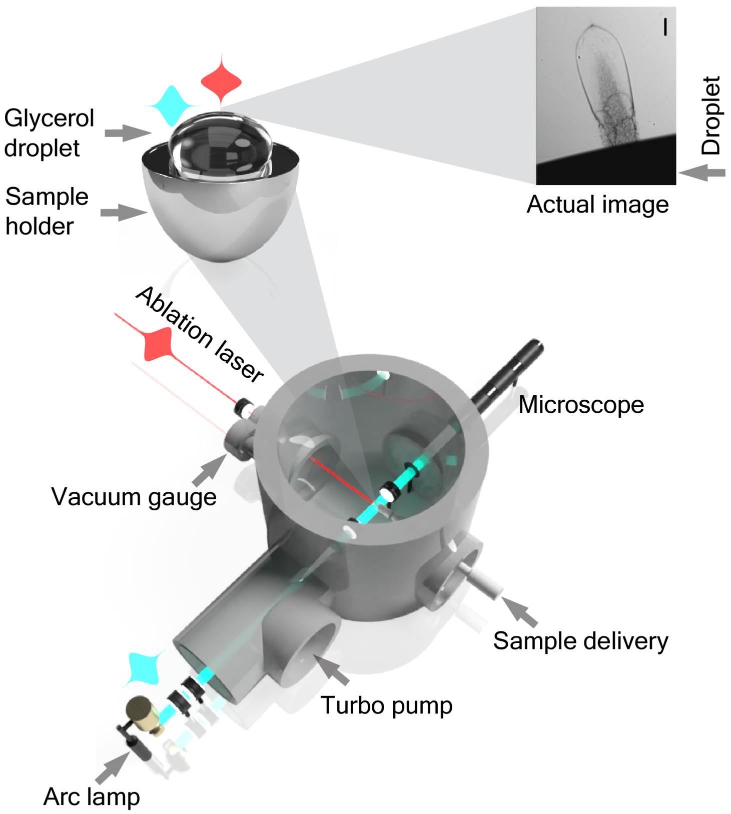

Fig. S6. Experimental setup and data collection. Detailed schematics of the time-resolved ablation measurement with vacuum housing and indicated beam paths. Red indicates the routing of the ablation laser, and cyan indicates the path of the white light pulse of the discharge lamp.

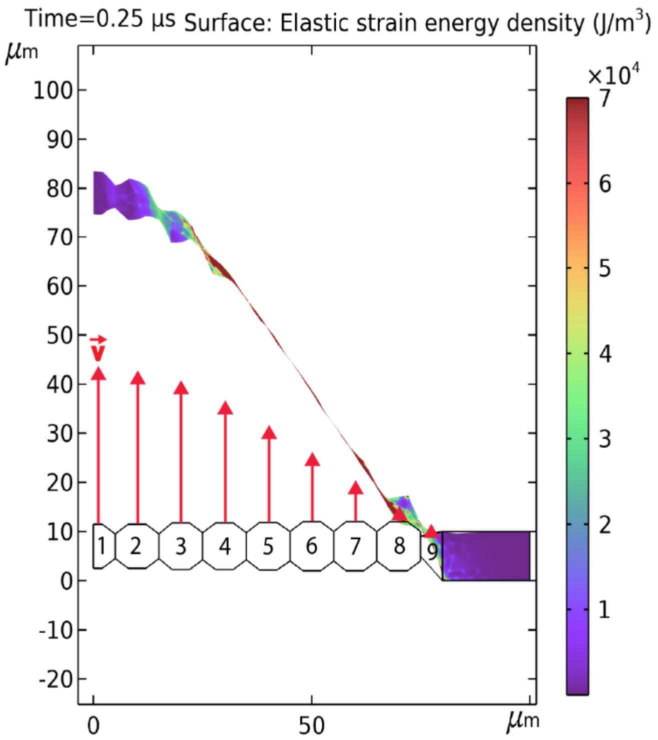

Fig. S7. The simulated early motion of the shell. The horizontal axis is the radius r; the vertical axis is the z-axis. The membrane comprises several toroidal sections, of which the inner eight have a hexagonal cross-section. The thickness is about 0.01 mm. The initial velocity (red arrows) of the different sections is indicated. Visible is the membrane at t=0 and t=250 ns. The colored sections show the elastic strain energy for the membrane at t=250 ns, where a neo-Hookean membrane becomes extremely thin in parts.

A

B

C

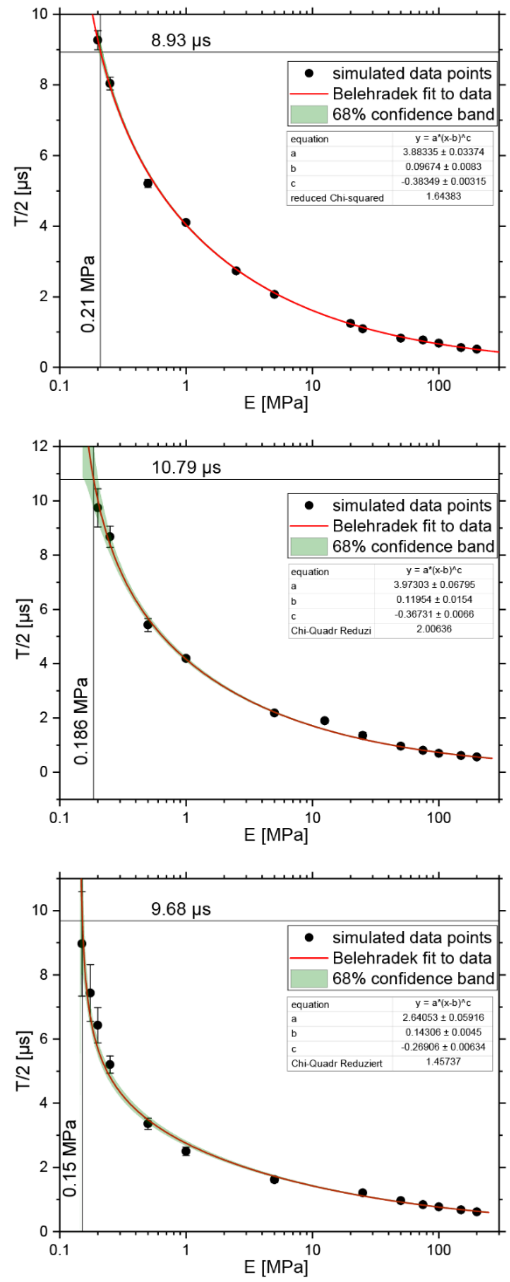

Fig. S8. Determination of Young's modulus of the shell. The dynamic of the shell, expressed by period length T of the motion, for different Young's moduli E and the three different laser fluences, mainly affecting the velocity distribution of the shell at $t = 0$. The data is fitted by $y = a(E - b)^c$ with fit parameters a, b, c. From the fitted function, Young's modulus (vertical line) is found from the experimental period times (horizontal line) according to Fig. 2 and Table S1. (**A**) Peak fluence of 240±11 mJ/cm^2. (**B**) Peak fluence of 206±9 mJ/cm^2. (**C**) Peak fluence of 174±8 mJ/cm^2

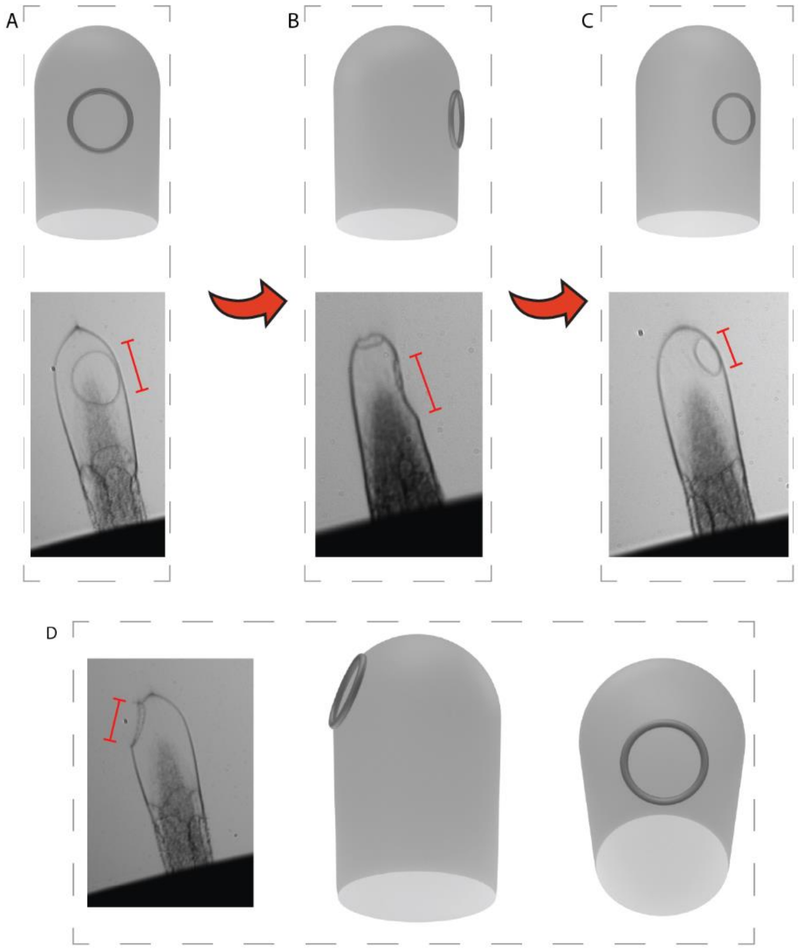

Fig. S9. Comparison of representative experimentally found projections of hole positions of circular fractures with abstracted representations. (A) Experimental and abstracted hole positions where the projection stays circular. A collection of similar shots does not provide any evidence of non-circular holes. (B) Hole position on the wall that is almost 90° rotated from the viewing direction, equivalent to a 90° clockwise rotation of A around the cylindrical symmetry axis S. (C) Hole position on the back wall facing away from the camera, equivalent to about 120° clockwise rotation of A around S. (D) Visualizes a case with a hole position on the left side wall along with its abstracted representation and different perspectives of D demonstrating the circular nature of the hole.

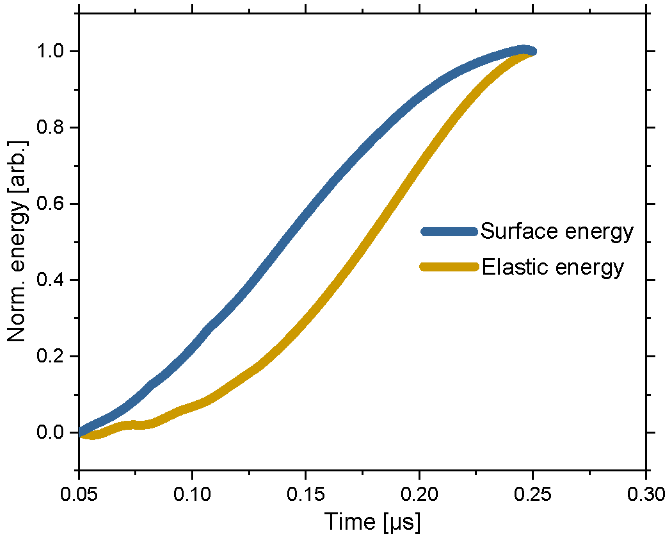

Fig. S10. Surface energy vs. Elastic energy. Normalized total energy for a simulated shell evaluated at a much higher Young's modulus of 170 MPa (0.2 MPa found in the experiment) where the simulation reaches the point at $E = 1$ where all initial kinetic energy of the shell is converted into a form of potential energy, either elastic energy or surface energy, depending on the scenario.

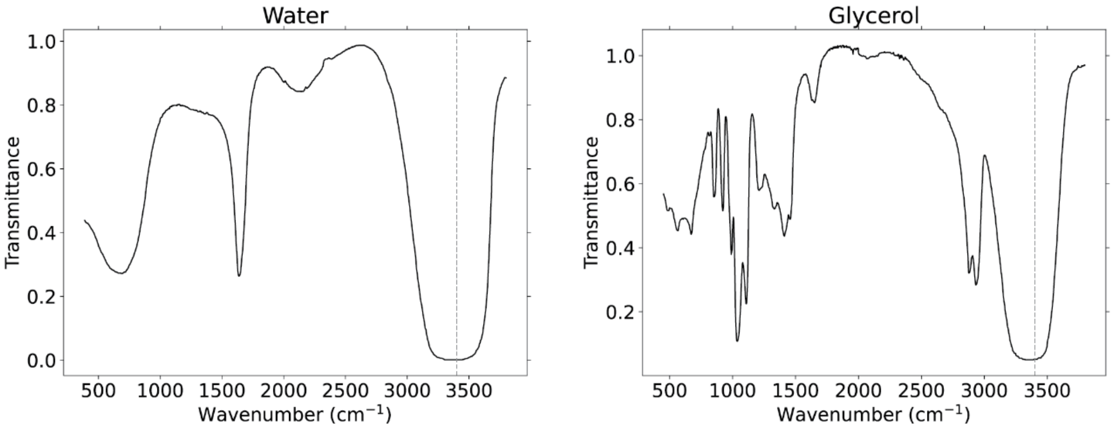

Fig. S11. IR spectrum for water and glycerol, with data from NCBI[1]. The dashed line indicates 3400 cm^{-1} wavenumbers. The figures are not normalized to each other.

[1] National Center for Biotechnology Information (NCBI)[Internet]. Bethesda (MD): National Library of Medicine (US), National Center for Biotechnology Information; [1988] – [cited 2023 Apr 05]. Available from: https://www.ncbi.nlm.nih.gov/

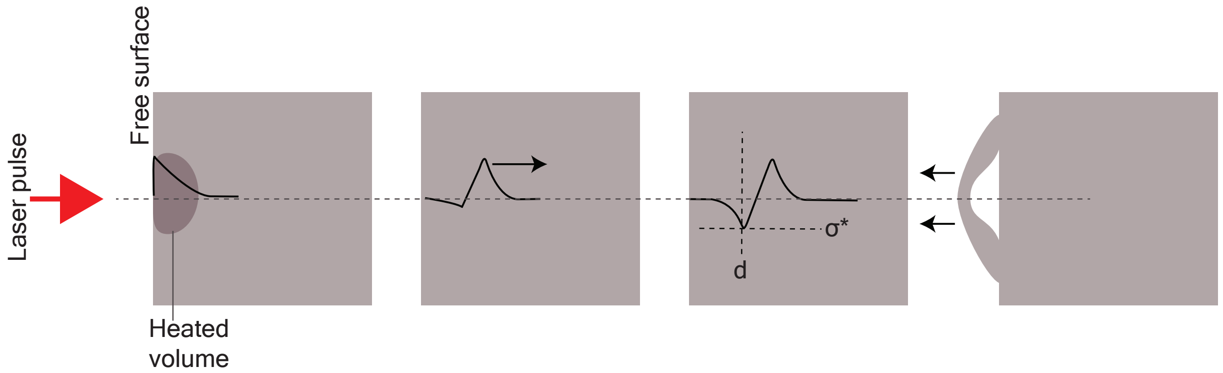

Fig. S12. Photomechanical spallation (front surface spallation) schematics. The thermal expansion of a volume below the free surface of a liquid leads to the generation of compressive stress waves. Upon this wave propagation to the free surface, a tensile component develops, and its amplitude increases gradually during propagation into the volume. Once the material's tensile strength (σ*) is reached at a depth (d), the material fractures. A spall layer is detached following this from the bulk. Adapted from Paltauf. et. al.(12).

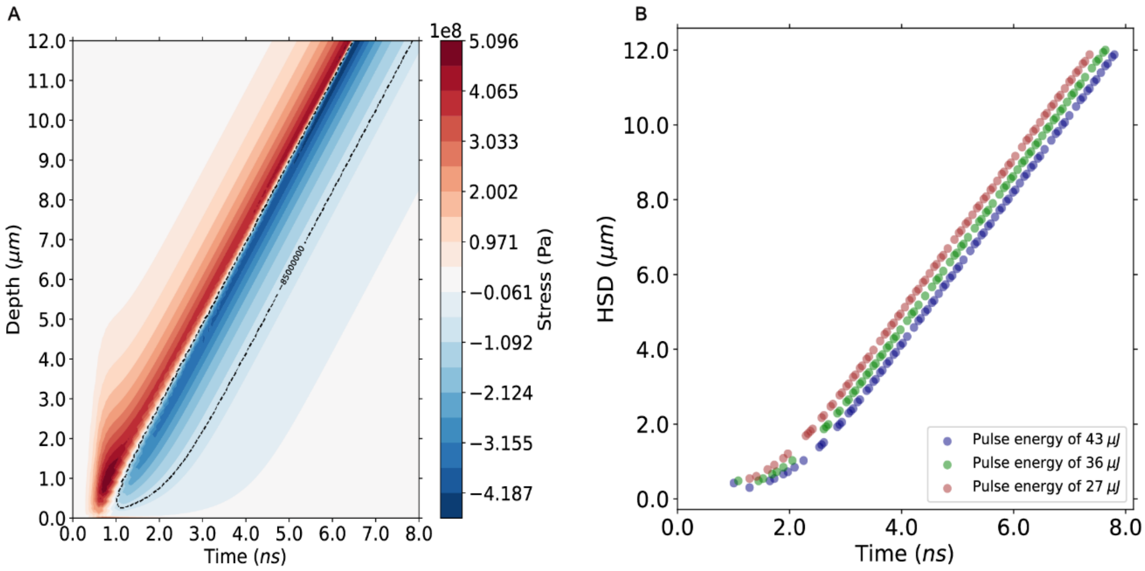

Fig. S13. Solution for the 1D thermoelastic wave equation in liquid glycerol. (**A**) One-dimensional solution (depth from the bulk surface) of the thermoelastic wave equation with compressive (red colors) and tensile stresses (blue colors) in liquid glycerol plotted as a function of time for a pulse energy of 43 μJ. The dotted black line indicates glycerol's reported dynamic tensile strength at 293 K. (**B**) Hypothetical spallation depth calculated for the three peak fluences expressed as pulse energies (calculated by integrating the fluence over the lateral area of the bubble).

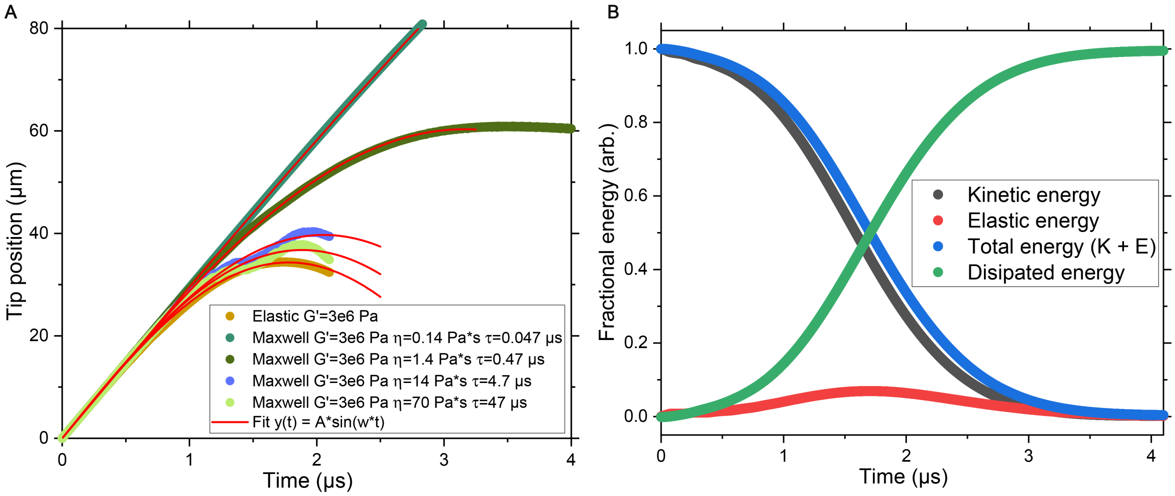

Fig. S14. (**A**) Tip position of mass point δm as a function of time for an elastic membrane (G'=3*10^6 Pa) and a membrane under the Maxwell rheological model for different viscosities. The data is fitted by functions of the form $y(t) = A * \sin(w * t)$. (**B**) Normalized distribution of energies (kinetic, elastic, dissipated, total) for a specific viscosity of η=1.4 Pa*s of the simulation in (**A**).

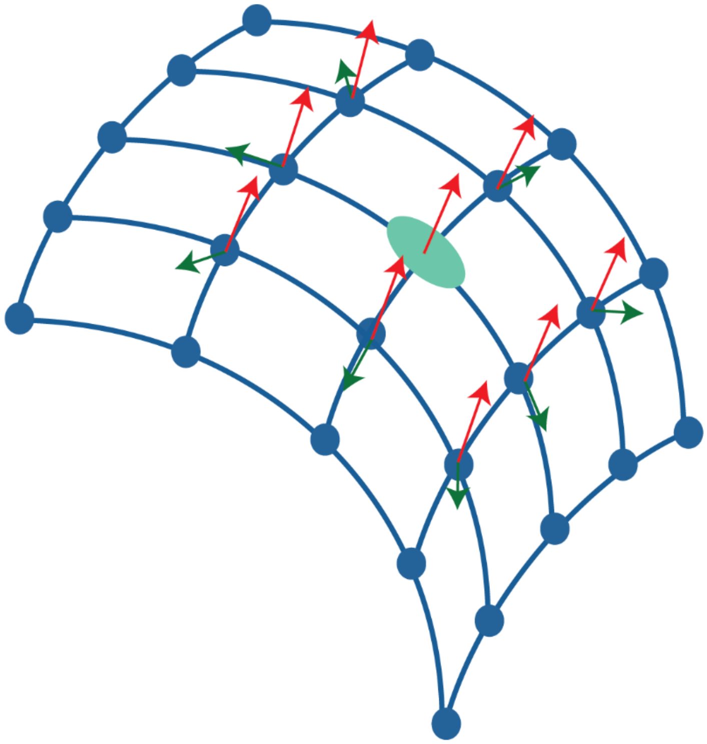

Fig. S15. Velocity components of mass points on the shell near the onset of a hole fracture (cyan). Red arrows represent the velocity component corresponding to the inflation of the shell and the green arrows represent the velocity components corresponding to the stretching of the surface.

Table S1. Parameters for the function $f(x) = A * sin(\frac{2\pi}{T} * t)$ derived for bubble tip motion described in Fig. 2.

**Fluence (mJ/cm^2)**	**A (µm)**	**T/2 (µs)**	**Dynamic Young's modulus (E, MPa)**
240±11	819±12	8.93±0.03	0.21±0.01
206±9	906±26	10.79±0.04	0.19±0.02
174±8	614±32	9.68±0.07	0.15±0.01

Table S2. Initial velocities of the shell segments for simulation according to Fig. S7 for the three different laser peak fluences.

Segment	Velocity [m/s] at 240±11 mJ/cm^2	Velocity [m/s] at 206±9 mJ/cm^2	Velocity [m/s] at 174±8 mJ/cm^2
1	288.4	263.5	200.6
2	282.9	258.5	192.7
3	260.5	238.0	178.9
4	223.9	204.6	157.2
5	177.5	162.2	127.1
6	126.4	115.5	90.8
7	75.2	68.8	53.4
8	28.1	25.7	22.3
9	6.9	6.3	5.0

Table S3. The storage modulus G' of glycerol at different frequencies.

**Frequency**	$\boldsymbol{G'}$	**Temperature**
f > 25 GHz	≈ 2.4 GPa	295 K (38)
14.7 MHz	23 MPa	295 K (39)
10 MHz	15.4 MPa	293 K (40)
0.1 MHz (this work)*	0.48 MPa	>295 K
f < 1 Hz	≈ 100 Pa	295 K (41)

*Refer to SI Appendix, Sec. I.

Movie S1 (separate file). Temporal evolution of the laser-induced bubble in glycerol. The bubble's temporal evolution for the first 3 µs under vacuum conditions of 10^{-3} mbar, initiated by an ablation fluence of 240±11 mJ/cm^2. The movie-like series of snapshots is not the temporal evolution of a single bubble but the temporally ordered sequence of individual snapshots of different ablation events recorded at different time points spanning over 3 µs.

SI References

1. R. K. Shori, A. A. Walston, O. M. Stafsudd, D. Fried, J. T. Walsh, Quantification and modeling of the dynamic changes in the absorption coefficient of water at λ = 2.94 μm. *IEEE J. Select. Topics Quantum Electron.* **7**, 959–970 (2001).
2. X. Fan, M. W. Little, K. K. Murray, Infrared laser wavelength dependence of particles ablated from glycerol. *Applied Surface Science* **255**, 1699–1704 (2008).
3. K. B. Beć, C. W. Huck, Breakthrough Potential in Near-Infrared Spectroscopy: Spectra Simulation. A Review of Recent Developments. *Front. Chem.* **7**, 48 (2019).
4. K. Franjic, R. J. D. Miller, Vibrationally excited ultrafast thermodynamic phase transitions at the water/air interface. *Physical Chemistry Chemical Physics* **12**, 5225 (2010).
5. Z. Chen, A. Bogaerts, A. Vertes, Phase explosion in atmospheric pressure infrared laser ablation from water-rich targets. *Applied Physics Letters* **89**, 041503 (2006).
6. Z. Chen, A. Vertes, Early plume expansion in atmospheric pressure midinfrared laser ablation of water-rich targets. *Physical Review E* **77** (2008).
7. B. Wu, From supercritical hydrodynamic expansion to explosive phase change: Thermodynamic evolution of water during its interaction with high-intensity infrared nanosecond-pulsed laser. *Journal of Applied Physics* **105**, 053502 (2009).
8. I. Apitz, A. Vogel, Material ejection in nanosecond Er:YAG laser ablation of water, liver, and skin. *Appl. Phys. A* **81**, 329–338 (2005).
9. K. Franjic, M. L. Cowan, D. Kraemer, R. J. D. Miller, Laser selective cutting of biological tissues by impulsive heat deposition through ultrafast vibrational excitations. *Opt. Express* **17**, 22937 (2009).
10. A. Vogel, V. Venugopalan, Mechanisms of Pulsed Laser Ablation of Biological Tissues. *Chemical Reviews* **103**, 577–644 (2003).
11. R. S. Dingus, R. J. Scammon, Grueneisen-stress-induced ablation of biological tissue in *Laser-Tissue Interaction Ii*, S. L. Jacques, Ed. (SPIE, 1991), pp. 45–54.
12. G. Paltauf, P. E. Dyer, Photomechanical Processes and Effects in Ablation. *Chemical Reviews* **103**, 487–518 (2003).
13. P. Ahmadi, A. Chapoy, R. Burgass, An investigation on the thermophysical properties of glycerol. *The Journal of Chemical Thermodynamics* **178**, 106975 (2023).

14. H. W. Tan, A. R. Abdul Aziz, M. K. Aroua, Glycerol production and its applications as a raw material: A review. *Renewable and Sustainable Energy Reviews* **27**, 118–127 (2013).

15. E. F. Carome, N. A. Clark, C. E. Moeller, Generation of acoustic signals in liquids by ruby laser-induced thermal stress transients. *Appl. Phys. Lett.* **4**, 95–97 (1964).

16. S. Liang, B. Lashkari, S. S. S. Choi, V. Ntziachristos, A. Mandelis, The application of frequency-domain photoacoustics to temperature-dependent measurements of the Grüneisen parameter in lipids. *Photoacoustics* **11**, 56–64 (2018).

17. H. Antlinger, *et al.*, Sensing the characteristic acoustic impedance of a fluid utilizing acoustic pressure waves. *Sensors and Actuators A: Physical* **186**, 94–99 (2012).

18. G. A. Carlson, H. S. Levine, Dynamic tensile strength of glycerol. *Journal of Applied Physics* **46**, 1594–1601 (1975).

19. P. Serra, A. Piqué, Laser-Induced Forward Transfer: Fundamentals and Applications. *Adv. Mater. Technol.* **4**, 1800099 (2019).

20. M. S. Brown, N. T. Kattamis, C. B. Arnold, Time-resolved dynamics of laser-induced micro-jets from thin liquid films. *Microfluid Nanofluid* **11**, 199–207 (2011).

21. R. Xiong, *et al.*, Bubble Formation Modeling During Laser Direct Writing of Glycerol Solutions. *Journal of Micro and Nano-Manufacturing* **3** (2015).

22. W. Wang, Y. Lin, Y. Huang, Modeling of Thermoelastic Stress Wave in Laser-Assisted Cell Direct Writing. *Journal of Manufacturing Science and Engineering* **133**, 024502 (2011).

23. D. D. Joseph, *Fluid Dynamics of Viscoelastic Liquids* (Springer New York, 1990).

24. D. Tammaro, *et al.*, Elasticity in Bubble Rupture. *Langmuir* **34**, 5646–5654 (2018).

25. G. H. McKinley, Dimensionless Groups For Understanding Free Surface Flows of Complex Fluids. HML Report Number 05-P-05 (2005).

26. D. D. Joseph, Historical perspectives on the elasticity of liquids. *Journal of Non-Newtonian Fluid Mechanics* **19**, 237–249 (1986).

27. D. D. Joseph, O. Riccius, M. Arney, Shear-wave speeds and elastic moduli for different liquids. Part 2. Experiments. *J. Fluid Mech.* **171**, 309 (1986).

28. A. B. Pandit, J. F. Davidson, Hydrodynamics of the rupture of thin liquid films. *Journal of Fluid Mechanics* **212**, 11 (1990).

29. G. Debrégeas, P.-G. de Gennes, F. Brochard-Wyart, The Life and Death of "Bare" Viscous Bubbles. *Science* **279**, 1704–1707 (1998).

30. G. Debrégeas, P. Martin, F. Brochard-Wyart, Viscous Bursting of Suspended Films. *Physical Review Letters* **75**, 3886–3889 (1995).

31. J. C. Bird, R. de Ruiter, L. Courbin, H. A. Stone, Daughter bubble cascades produced by folding of ruptured thin films. *Nature* **465**, 759–762 (2010).

32. N. Savva, J. W. M. Bush, Viscous sheet retraction. *J. Fluid Mech.* **626**, 211–240 (2009).

33. D. Tammaro, *et al.*, Flowering in bursting bubbles with viscoelastic interfaces. *Proceedings of the National Academy of Sciences* **118** (2021).

34. C. W. Barney, *et al.*, Cavitation in soft matter. *Proceedings of the National Academy of Sciences* **117**, 9157–9165 (2020).

35. A. T. Oratis, J. W. M. Bush, H. A. Stone, J. C. Bird, A new wrinkle on liquid sheets: Turning the mechanism of viscous bubble collapse upside down. *Science* **369**, 685–688 (2020).

36. M. M. Villone, G. D'Avino, E. D. Maio, M. A. Hulsen, P. L. Maffettone, Modeling and simulation of viscoelastic film retraction. *Journal of Non-Newtonian Fluid Mechanics* **249**, 26–35 (2017).

37. O. Teschke, D. Mendez Soares, J. F. Valente Filho, Floating liquid bridge tensile behavior: Electric-field-induced Young's modulus measurements. *Appl. Phys. Lett.* **103**, 251608 (2013).

38. C. Klieber, *et al.*, Mechanical spectra of glass-forming liquids. II. Gigahertz-frequency longitudinal and shear acoustic dynamics in glycerol and DC704 studied by time-domain Brillouin scattering. *The Journal of Chemical Physics* **138**, 12A544 (2013).

39. R. Piccirelli, T. A. Litovitz, Ultrasonic Shear and Compressional Relaxation in Liquid Glycerol. *The Journal of the Acoustical Society of America* **29**, 1009–1020 (1957).

40. A. Bund, G. Schwitzgebel, Viscoelastic Properties of Low-Viscosity Liquids Studied with Thickness-Shear Mode Resonators. *Anal. Chem.* **70**, 2584–2588 (1998).

41. L. Noirez, P. Baroni, Revealing the solid-like nature of glycerol at ambient temperature. *Journal of Molecular Structure* **972**, 16–21 (2010).
