# Supplementary figures and images for: Rubber-like elasticity in laser-driven free surface flow of a Newtonian fluid
Source: Proc Natl Acad Sci U S A. 2023 Jun 26;120(27):e2301956120. doi: 10.1073/pnas.2301956120 (PMC10319024; doi:10.1073/pnas.2301956120)

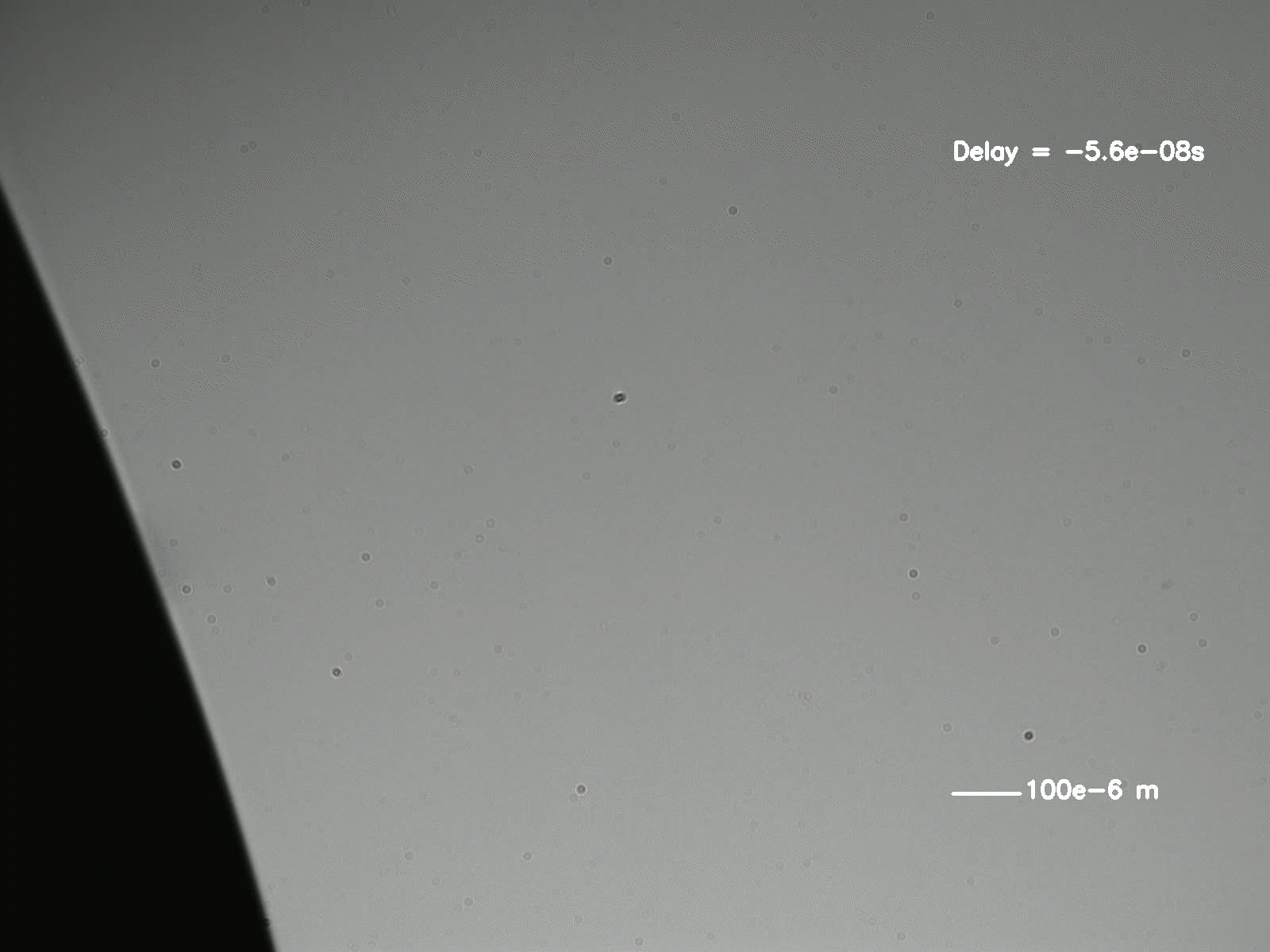

Supplement: Movie S1. — Temporal evolution of the laser-induced bubble in glycerol. The bubble’s temporal evolution for the first 3 μs under vacuum conditions of 10−3 mbar, initiated by an ablation fluence of 240±11 mJ/cm2. The movie-like series of snapshots is not the temporal evolution of a single bubble but the temporally ordered sequence of individual snapshots of different ablation events recorded at different time points spanning over 3 μs. [file pnas.2301956120.sm01.gif]
